# Johne's Disease in Dairy Cattle: An Immunogenetic Perspective

**DOI:** 10.3389/fvets.2021.718987

**Published:** 2021-08-26

**Authors:** Sanjay Mallikarjunappa, Luiz F. Brito, Sameer D. Pant, Flavio S. Schenkel, Kieran G. Meade, Niel A. Karrow

**Affiliations:** ^1^Department of Animal Biosciences, Centre for Genetic Improvement of Livestock, University of Guelph, Guelph, ON, Canada; ^2^Department of Animal Sciences, Purdue University, West Lafayette, IN, United States; ^3^Graham Centre for Agricultural Innovation, Charles Sturt University, Wagga Wagga, NSW, Australia; ^4^School of Agriculture and Food Science, University College Dublin, Dublin, Ireland

**Keywords:** cattle, MAP infection, Johne's disease, paratuberculosis, disease resistance

## Abstract

Johne's disease (JD), also known as paratuberculosis, is a severe production-limiting disease with significant economic and welfare implications for the global cattle industry. Caused by infection with *Mycobacterium avium* subspecies *paratuberculosis* (MAP), JD manifests as chronic enteritis in infected cattle. In addition to the economic losses and animal welfare issues associated with JD, MAP has attracted public health concerns with potential association with Crohn's disease, a human inflammatory bowel disease. The lack of effective treatment options, such as a vaccine, has hampered JD control resulting in its increasing global prevalence. The disease was first reported in 1895, but in recognition of its growing economic impact, extensive recent research facilitated by a revolution in technological approaches has led to significantly enhanced understanding of the immunological, genetic, and pathogen factors influencing disease pathogenesis. This knowledge has been derived from a variety of diverse models to elucidate host-pathogen interactions including *in vivo* and *in vitro* experimental infection models, studies measuring immune parameters in naturally-infected animals, and by studies conducted at the population level to enable the estimation of genetic parameters, and the identification of genetic markers and quantitative trait loci (QTL) putatively associated with susceptibility or resistance to JD. The main objectives of this review are to summarize these recent developments from an immunogenetics perspective and attempt to extract the principal and common findings emerging from this wealth of recent information. Based on these analyses, and in light of emerging technologies such as gene-editing, we conclude by discussing potential future avenues for effectively mitigating JD in cattle.

## Introduction

### Johne's Disease—Global Context, Economic, and Zoonotic Relevance and Control

The Food and Agriculture organization (FAO) estimates that in order to meet the growing demand from a world population projected to reach over 9 billion, annual meat production will need to increase by 41 million tons over 2019 production levels to 376 million tons by 2030 (1). In addition, according to International Farm Comparison Network (IFCN), growth in global milk production be required to increase by 35% by 2030 (2).

Leading the response to this demand is the bovine industry, and a number of recent changing trends in dairy production across the globe have been identified, with significant increases in the size of dairy herds (3), and a sectoral expansion in the EU after the abolition of milk quotas (4). These increasing cattle numbers and production levels, and other factors like recent changes toward increased animal housing in parts of the world, will exacerbate issues associated with the control of infectious diseases (5). A key feature of successful farm enterprises of the future will be efficiency of production, and multiple infectious diseases not only threaten sectoral efficiency and sustainability, but also have serious animal welfare and public health implications (6). There is a growing appreciation of the need to control infectious diseases at source, and infections with zoonotic potential are of particular concern. The advent of the “One Health” approach to achieve optimal health among humans, animals and environment is a major step in the efforts to control infectious and zoonotic diseases (7).

The focus of this review is on one such disease with serious animal production, welfare, and potential human health implications named Johne's disease (JD) or paratuberculosis. JD is a chronic progressive intestinal inflammatory disease caused by infection with *Mycobacterium avium* subsp. *paratuberculosis* (MAP) in cattle. The disease is named after a German pathologist, Heinrich Albert Johne, and was first reported in cattle in 1895 (8). JD pathogenesis is characterized by a long latent sub-clinical phase lasting for years, followed by clinical phase, where overt signs such as chronic diarrhea, emaciation, and decreased milk production and fertility are exhibited by infected animals before eventually leading to death (9).

JD is now a globally prevalent contagious disease with major economic and welfare implications on the cattle industry. In a recent survey involving 48 countries, authors reported a herd-level prevalence of JD ranging between 1 and >40%; and the within-herd prevalence ranging between 1 and 15% in dairy cattle alone (10). Prevalence of JD is not just limited to livestock, as MAP has also been isolated from wild ruminants as well as non-ruminants suggesting their possible role as reservoir hosts (11). Ruminant species such as bison (11), white-tailed deer (12), red deer (13), elk (14), and non-ruminant species such as wild rabbits, foxes, guanacos (15), and even primates such as mandrills and macaques (16), have been shown to harbor MAP illustrating the broad host species adaptation of this mycobacteria. MAP has also been isolated from free-living amoebae suggesting their under-appreciated role as vectors in water-borne MAP transmission (17). This broad range of host species could account for widespread transmission of MAP making it difficult to control JD across the globe.

MAP is shed from infected hosts into the environment through feces, therefore, the fecal-oral route is considered the primary mode of transmission. Neonatal calves, and calves <6 months of age, are highly susceptible to MAP infection (18), via their consumption of contaminated colostrum, waste milk, and/or feed contaminated with feces containing MAP (19, 20). Other routes of MAP transmission have also been documented including intra-uterine transmission from dam to calf (21), potential transmission through semen from infected bulls (22), and the bio-aerosol route (23).

As a result of its success as a pathogen, MAP infection is responsible for significant negative impacts on dairy cattle economics worldwide. Herd-level economic losses associated with JD in U.S. dairy operations was estimated at $200-$250 million dollars annually (24). In Canada, estimated loss due to JD is $49 CAD per cow per year (25). For a cow in a JD affected herd in Ireland, a profit margin reduction between €168 and €253 was estimated (26), while in Australia, it was estimated to be AUD 44.84 per cow/year (27). Although these are per cow estimates, the losses incurred amount to millions in lost revenue when the overall affected population is considered. These losses are due to decreased milk production in the infected herds, increased mortality and premature culling of MAP infected animals, reduced fertility, reduced slaughter value as JD is associated with weight loss, increased management costs, and diagnostic and veterinary costs aimed at reducing the incidence of JD (28). Further, initial reports have speculated that MAP co-infection as a potential reason for thwarted bovine tuberculosis (bTB) eradication schemes in United Kingdom, as chronically bTB infected herds have increased risk of having positive MAP infection status as opposed to non-chronically infected herds (29).

Another serious implication of MAP is its potential zoonotic association with Crohn's disease (CD), an inflammatory bowel disorder in humans (30). The basis for this zoonotic link stems from studies reporting detection of MAP from patients suffering from CD (31–35) along with reports of remission of clinical symptoms in CD patients treated with antimicrobial drugs (36). Despite this, the zoonotic nature of MAP is still deemed debatable and will remain such until conclusive evidence is drawn explaining the cause-effect mechanism involved between MAP and CD (37, 38). Of particular concern for the globally expanding dairy sector is the reports of MAP being detected in pasteurized milk and dried milk-based products, which has sparked MAP public health concern debates (39). Together, these two elements of heat resilience and association with CD makes MAP one of the most potentially devastating infectious disease agents for the global dairy industry.

Currently, there is no cure for MAP, and JD control across the globe has proven difficult. The first contributory issue is that it is challenging to fight MAP infection when it is so difficult to detect. As JD is a contagious infectious disease, its accurate diagnosis during early stages is critical to limit MAP spread and infection within and across herds. JD diagnosis is based on immune assays like ELISA that detect MAP-specific antibodies in milk or serum, and by tests that detect MAP in feces or tissues either by culture, or polymerase chain reaction (PCR). However, these tests are limited in their ability to diagnose early stages of MAP infection because of their reduced sensitivities during subclinical stages where antibody levels and fecal MAP shedding are low (40). Adding to this, MAP culture techniques are highly specific, but lack sensitivity during intermittent shedding of JD, plus MAP culture requires a long turnaround time to confirm positivity (41). Milk/serum ELISA is a commonly used diagnostic method because it is cost-effective, simple to perform, and has a quick turnaround time compared to MAP culture. However, the drawback of this method is its low sensitivity (30%), which may not allow for detection during the early phase of infection when the antibody response is minute (40).

Prevention of JD is the most preferable option, but currently no efficacious vaccine exists to confer protection against MAP. Inactivated MAP vaccines tested in cattle have so far only been effective at reducing fecal shedding and tissue colonization, and are unable to eliminate MAP infection (42, 43), and efforts to produce a vaccine that can prevent MAP infection and/or confer protective immunity are still ongoing (44).

With the absence of vaccines and treatment options, the current control measures to reduce JD incidence are management-based; including employing a “test and cull strategy” to remove MAP infected animals from the herd (45), and enhancing on-farm biosecurity and surveillance measures to prevent MAP transmission within and between herds (46). In this regard, several countries have adapted voluntary JD eradication programmes (47). Genetic regulation of the host response to MAP infection is also being extensively studied in order to explore potential genetic selection strategies to enhance resistance of dairy cattle to JD (48).

Overall, JD continues to seriously plague the cattle industry worldwide. Understanding of host immune response to MAP infection and its genetic regulation in cattle has been made possible by diverse research studies over the years. We now know that response to MAP infection is complex and heritable (49), leading to differences in clinical presentations of JD between individuals. These findings have opened up potential new avenues for early disease diagnosis and implementation of genetic and genomic selection schemes for breeding more JD resistant animals. Furthermore, the genetic regulation of the same has been studied by estimating genetic parameters and identifying genetic markers influencing JD susceptibility and resistance in cattle. The main objectives of this review are to highlight the key findings from an immunogenetic perspective, potential mitigation strategies through selective breeding, and to highlight knowledge-gaps that future research efforts could aim to address to help advance our progress toward the control of JD.

## Understanding Map Infection

### JD Pathogenesis

**Figure 1:**
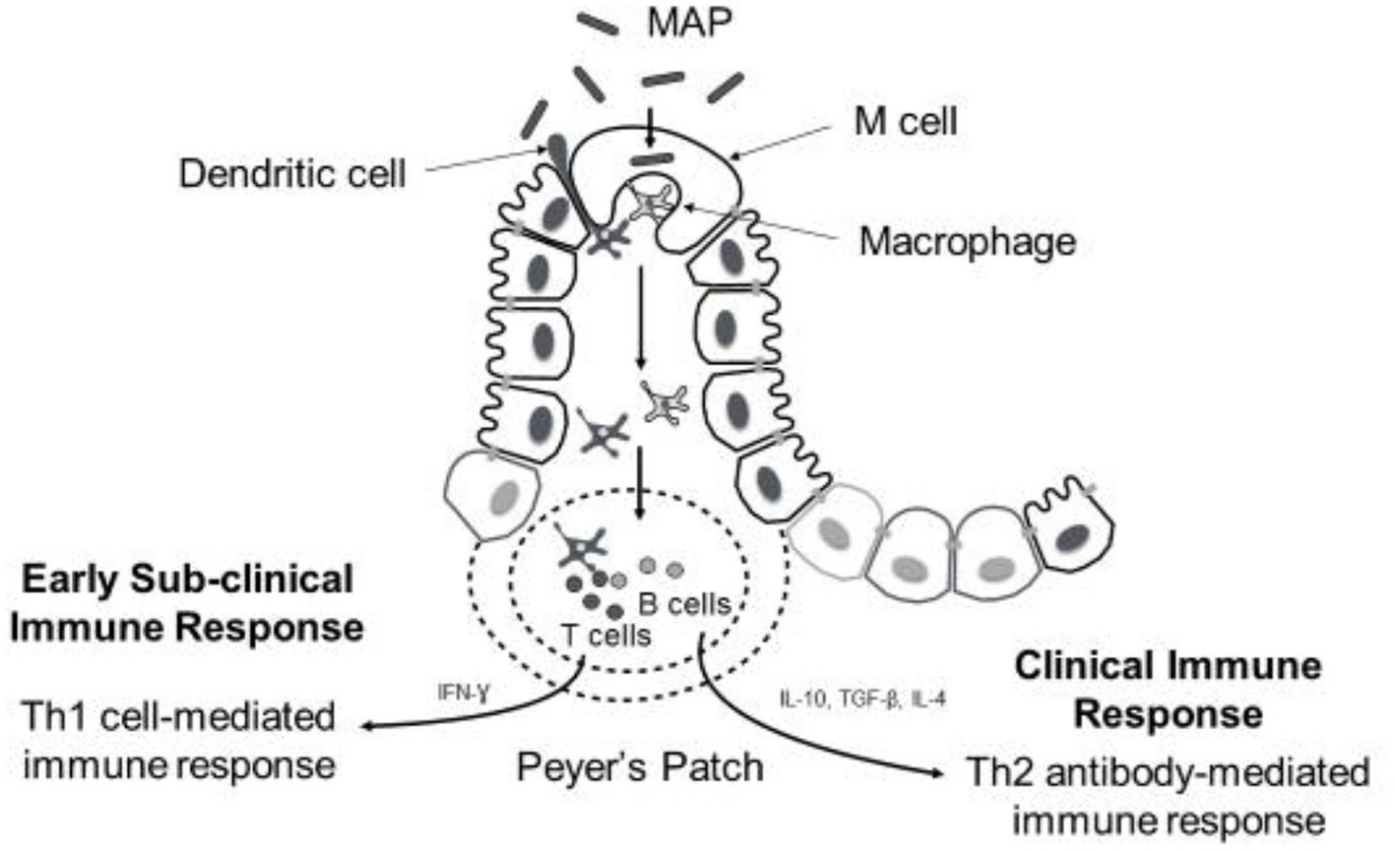
Diagrammatic representation of MAP uptake and immune response during disease progression. Following oral ingestion of MAP, M cells facilitate uptake of MAP across mucosal epithelium into submucoal gut-associated lymphatic tissue (peyer's patches). Here MAP is phagocytozed by macrophages (sometimes dendritic cells can sample MAP from intestinal lumen directly). Early immune response against MAP infection involves activation of macrophages and T-cells to promote Th1 cell-mediated immune response. Predominance of cell-mediated immune response is observed during subclinical stage of the disease; however, the onset of clinical signs coincides with shift from Th1- to- Th2 antibody-mediated immune response.

Despite the evidence for an extensive environmental reservoir of MAP, infection levels in dairy cattle are relatively low at a national level, implying that possibly innate immune processes and the genetic background of the host may play a determining role in resistance to infection. Resilience to MAP infection is seen in cattle, and it has been reported that only fraction of infected animals progressing to clinical JD (50). The pathogenesis of JD follows various stages and is broadly classified into early, sub-clinical and late infection phases. The early stage encompasses interactions between MAP, which is an obligate intracellular bacterium, and innate immune cells called macrophages. Following ingestion by the host, MAP translocates into gut-associated lymphatic tissues (GALT) where it is phagocytosed by macrophages. Translocation across the mucosal epithelium is facilitated by specialized intestinal epithelial cells such as M cells and enterocytes through fibronectin-dependent mechanisms (51–54), and likely also by motile dendritic cells that can directly sample the pathogen from the intestinal lumen and migrate to draining lymph nodes to carry out antigen presentation (55) (Figure 1). Pattern recognition receptors (PRRs) on the macrophage surface, such as complement receptors (56), mannose receptors (57, 58), β-integrin receptors such as CD11a and CD18 (59), and CD14 receptors (60), are shown to mediate MAP recognition and phagocytosis. When fully activated, macrophages are capable of exerting their microbicidal defense against MAP such as MAP ingestion and creation of a phagosome. However, similar to other mycobacteria spp., MAP has evolved several strategies to survive within host cells like macrophages and disseminate infection within the host (61). These include inhibiting phagosomal maturation (62), interfering with macrophage apoptosis and phagosome acidification (63), evading antigen presentation by macrophages by down-regulating the expression of MHC molecules (64), and regulating different signal transduction pathways (65, 66). Successful establishment of MAP within host cells in the early stage gives way to the latent subclinical phase that can last for 2–5 years (67). During the subclinical phase, no display of overt clinical signs is noticed in the infected animal making JD diagnosis difficult, although intermittent fecal shedding of MAP is often detected later leading to MAP contamination and spread within herds. During both the early and subclinical phases, the host inflammatory response induces localized granuloma formation aimed at sequestering MAP-infected macrophages to contain infection (67). However, as disease progresses, further dissemination of MAP infection leads to severe intestinal inflammatory granulomatous lesions and subsequent onset of the clinical phase of JD, which is characterized by the previously described clinical symptoms (68). These clinical signs are a result of the immunopathology associated with granulomatous lesions, which include thickening of intestinal wall leading to malabsorption, chronic diarrhea and profuse protein losing enteropathy (9, 68). Diagrammatic representation of MAP uptake and immune response is presented in Figure 1.

### Host Immune Response

The immune response to MAP infection in cattle involves a complex interplay between host and the pathogen highlighted by activation of different immune cells by numerous cytokines and co-stimulatory molecules during different stages of JD pathogenesis. Phagocytosis of MAP by macrophages is followed by an early cell-mediated immune response. This includes activation of immune cells such as γδ T cells, antigen processing and presentation by macrophages, and the activation of CD4^+^ T cells whose effector function has a great bearing on the outcome of MAP infection in the host particularly during early stages (67, 69). CD4^+^ T cell subtypes are responsible for steering the cell-mediated immune response against intracellular MAP. Activated macrophages secrete IL-12 and chemokines that recruit CD4^+^ T cells to the GALT. CD4^+^ T cells further recognize MAP antigenic determinants presented by macrophages via MHC II molecules and in turn secrete IFN-γ, which enhances macrophage effector killing functions (67). IFN-γ further acts on macrophages to secrete more IL-12, which stimulates proliferation of more CD4^+^ T cells leading to enhanced inflammatory changes and polarizes a Th1 cell-mediated immune response (67, 69). The IFN-γ ELISA, a diagnostic tool used to detect sub-clinical stages of JD is based on measurement of production of IFN-γ by MAP-stimulated peripheral blood mononuclear cells (PBMCs) (70). The ability of IFN-γ to enhance the anti-mycobacterial activity of macrophages against intracellular MAP is well-documented; it has been reported that IFN-γ induces nitric oxide synthesis and phagosome maturation in MAP-infected macrophages, which negatively affects the intracellular survival of MAP (71–73). Although protective in nature, prolonged production of IFN-γ at the infection site also leads to chronic inflammation and contributes to immunopathology associated with JD (74).

In a recent review, the potential inflammatory role of the IL-17a cytokine secreted by Th17 cells, and IL-23 which activates Th17 cell cytokine production, was also highlighted in JD pathogenesis (75). IL-17 mediates recruitment of neutrophils to mycobacterial infection sites (76), and increased levels of IL-17a were noticed in the early stage lesions in the ileum of MAP-infected animals (77). While the early induced immunological responses will influence the disease outcome in the infected animal (78, 79), MAP has evolved strategies to redirect the early immune response to favor its survival.

In recent years, the role of γδ T cells during early infection stages of MAP has been extensively studied. γδ T cells constitute about 40% of circulating peripheral blood mononuclear cells (PBMC) in calves and 10–15% in adult cattle and are thought to be a link connecting the bovine and human innate and adaptive immune responses during mycobacterial infections (80). Based on expression of scavenger receptor workshop cluster 1 (WC1), two different subsets of γδ T cells have been defined in cattle; WC1^+^ and WC1^−^ (81). While WC1^+^ are predominantly distributed in peripheral blood, WC1^−^ are primarily found in spleen, uterus, intestinal mucosa, and lymph nodes (81, 82). Early recruitment of γδ T cells and their presence in MAP-induced lesions have been reported in experimental calves injected with live MAP inoculum and MAP-whole cell vaccine suggesting that they play a role in granuloma formation (83). Given the relative abundance of γδ T cells in the gut mucosa, their role as an innate source of IFN-γ and IL17α (84), and their ability to influence differentiation and maturation of monocytes (85), impact MAP viability (86), and initiate granuloma formation (83), γδ T cells likely play a critical role during the early stages of MAP infection.

Predominance of a Th1-mediated immune response is observed during the early sub-clinical stage as extensive proliferation of CD4^+^ T cells with the resultant increased expression of pro-inflammatory cytokines like IFN-γ is detected in the ileal tissue of sub-clinically infected animals (87, 88), in PBMCs (89), and PBMCs stimulated with MAP whole-cell sonicate (90). However, as the disease progresses, the Th1 cell-mediated immune response wanes coupled with an increased Th2 antibody-mediated response (67) and subsequent detectable levels of the IgG1 subclass of anti-MAP antibodies (91). The exact reason behind this Th1 to Th2 transition, and the time at which it takes place, is currently unknown, but it corresponds with onset of clinical disease. While earlier studies have reported the role of IL-10 producing regulatory T cells (CD4^+^ CD25^+^) in the switch to Th2 antibody response (92, 93), other reports have also suggested T cell unresponsiveness (77, 94), and loss of antigen-specific CD4^+^ T cells (95), as possible reasons for Th1 to Th2 switch. With a transition in the nature of immune response, predominant up-regulation of anti-inflammatory cytokines such as IL-10, TGF-β, and IL-4 in PBMCs, the ileum and associated lymph nodes tissues is also observed in naturally infected cattle (64–66). IL-4 is a Th2 polarizing cytokine that promotes activation and proliferation of B-cells and antibody production by plasma cells, which correlates with detectable antibody levels during the clinical stage (96). The immunoregulatory effect of IL-10 in suppressing IFN-γ production and favoring MAP viability is well-documented (88, 97, 98). The clinical stage is also characterized by constant shedding of MAP in the feces, milk, and colostrum (19, 20), thereby increasing the risk of MAP transmission within the herd.

### *In vitro* Models

Our knowledge of the host immune response to MAP infection has been acquired through the use of *in vitro* and *in vivo* experimental MAP challenge studies, and in some cases by studies that used naturally MAP-infected animals. Several *in vitro* models concerned with MAP infection have contributed immensely to our knowledge of MAP interaction with various immune cells and immortal cell lines, and in explaining host immune response and JD pathogenesis. Since macrophages are the main effector cells of JD pathogenesis, they are the predominant cells used in *in vitro* cell culture studies to study host-MAP interaction. The *in vitro* cell culture-based MAP-mononuclear phagocytic cell interaction studies have enabled our current understanding of the role of macrophage receptors in MAP phagocytosis (59), the ability of MAP to promote its survival by inhibiting processes such as macrophage apoptosis (63), phagosomal acidification (99) and maturation (62), and MAP's ability to inhibit signaling pathways such as JAK-STAT (66), CD40L-CD40 signaling (65), as well as to suppress monocyte microbicidal activity (99, 100). For instance, by developing a co-culture model involving the bovine mammary epithelial cell line (MAC-T) and bovine monocyte-derived macrophages (MDM), followed by challenge with MAP, Lamont et al. showed how phagosome acidification in MAC-T cells and subsequent Ca^+2^-dependent IL-1β secretion initiates recruitment of macrophages to the apical side of MAC-T cells and the entry of MAP into macrophages (100). Albeit this was shown *in vitro*, a similar mechanism could be in play to establish MAP infection at the intestinal mucosal level of MAP-infected animals.

The role of γδ T cells in the context of early MAP infection in cattle has also been explored extensively. By developing a bovine γδ T cell-MDM co-culture *in vitro* model, Baquero et al. (84) showed co-culturing of primary WC1^+^ γδ T cells in direct contact with MAP-infected MDMs led to decreased MAP viability and increased MHC-I expression on MDM. It was also shown that WC1^+^ and WC1^−^ γδ T cells impacted the function of monocytes during early MAP infection through their involvement in the differentiation and maturation of monocytes and dendritic cells (85). Decreased MAP viability was also observed when WC1^+^ and WC1^−^ γδ T cells from young calves were separately co-cultured in direct contact with MAP-infected MDM (86).

Cell culture tools and techniques are continually evolving. The recent revolution in genetic engineering using CRISPR/Cas9 has rendered efficient modification and editing of genes. The translation of such techniques is attainable using bovine cell lines that can be further tested to study gene function, gene interaction, and signaling pathway analysis. In the context of MAP infection, gene edited cell lines offer a sound platform to validate biological relevance of JD candidate genes, and related studies are now showing up in the literature (101). Recently, *in vitro* modeling of intestinal human diseases using 3D-intestinal organoids has gained prominence (102), and similar approaches may also be adapted to study JD in cattle.

### MAP Animal Infection Models

Understanding JD pathogenesis and the early infection of MAP at the host tissue level has been made possible by various MAP bovine infection models. By creating ligated ileal loops in calves followed by inoculation with MAP, Momotani et al. (51) demonstrated MAP uptake by ileal M-cells and further entry into subepithelial and intraepithelial macrophages within 5 h of inoculation. They also showed enhanced MAP uptake when anti-MAP serum from MAP infected cows was added to the inoculum. Using similar ligated ileal loop model, Khare et al. (103) further studied early morphological lesions and changes in the expression of several immune-related transcripts upon MAP inoculation. Within 30 min of inoculation, they observed MAP entry into ileal mucosa, and within 4 h of inoculation, increased expression of chemokines IL-8 and chemokine monocyte chemotactic peptide (MCP-1/CCL2) was seen with concurrent increased influx of migratory neutrophils and monocytes in submucosal lamina propria. The expression of pro-inflammatory genes (*IL-1*β, *TNF-*α, *IL-6*, and *IL-15*) was also high 4 h post-inoculation. After injecting MAP into ileal lumen, Wu et al. (104) studied the immune response during the following 9 months post MAP inoculation. The authors reported persistent infection of ileum and mesenteric lymph nodes throughout the duration of the study with a predominant Th1 type immune response, and the absence of fecal shedding and humoral response. Using the same model, they were also successful in differentiating the virulence of mutant and wild-type MAP strains, wherein the mutant strain failed to establish infection. After directly inoculating MAP into ileum using an ileal cannulation model, Allan et al. (105) observed a similar immune response as observed after oral challenge (106). In another oral MAP experimental challenge, early transcriptomic changes in PBMCs in MAP exposed animals indicated consistent changes in the regulation of antigen presenting pathway and processing genes and downregulation of lipid pathway associated and anti-bacterial defense genes (*CD38*) (107, 108). By directly inoculating MAP into ileocecal Peyer's patches, Plattner et al. (96) induced consistent intestinal MAP infection as evidenced by dose-dependent histopathological lesions, MAP culture positivity from tissues along with intermittent fecal shedding. The same model was also used to assess efficacy of future MAP vaccines and drug supplements (109). In another model, surgical isolation of an ileal segment of calf and its inoculation with MAP enabled persistent enteric localized MAP infection leading to the recruitment of macrophages, dendritic cells, CD8^+^, and γδ T cells in lamina propria (110). Furthermore, increased expression of cytokines TNF-α and IFN-γ by lamina propria leukocytes was also observed. The above described models offer tremendous insight into early host-pathogen interaction dynamics post MAP challenge. But the chronic nature of JD means that monitoring clinical progression of disease in infected animals is challenging and would require conducting longitudinal experimental trials that could span several years (>5 years). However, as observed by Begg et al. in their longitudinal study, not all the infected animals will progress into clinical forms as some recover from infection (111). Such animals are particularly intriguing as models to study immunogenetics aspect associated with resilience to MAP infection.

### Genomic Approaches to Identify Putative Biomarkers

While the host response at a cellular level to MAP infection has been investigated comprehensively, the molecular regulation of the immune response is still yielding valuable insights that offer resolution into the evolutionary battle between the host and MAP. Next-generation sequencing (NGS) technology-based RNA-sequencing (RNA-seq) studies are commonly employed to identify and quantify the expression of differentially regulated genes (DEGs). These DEGs are further subjected to bioinformatic analyses that enable identification of up- and down-regulated biological pathways in the context of MAP infection, and thereby generate better understanding of the biological processes associated with MAP infection and the host immune response. For instance, RNA-seq has been successfully used to profile transcriptome expression in intestinal tissues (e.g., ileal tissue, ileocecal valve), salivary glands, PBMC, and macrophages challenged with MAP.

In order to understand gene regulation at the primary site of MAP infection and how it varies between uninfected cows (control) and naturally infected subclinical and clinical JD cow groups, Hempel et al. (112) compared the ileocecal valve (ICV) transcriptome profile between the respective groups, and their results suggested enrichment of different pathways between groups. While differentially expressed genes between the clinical and control group influenced immunological pathways like immune cell receptor signaling and apoptosis, genes related to metabolism were differentially expressed between subclinical JD and the control group. Moreover, comparison between clinical and subclinical JD cows identified genes with a role in chemotaxis, leukocyte migration, complement, and coagulation pathway. Interestingly, in another comparative transcriptome study involving naturally infected cows with JD-associated histopathological lesions and control cows with no lesions, the CXCL8/IL8 signaling pathway, which plays a role in neutrophil recruitment, was found to be differentially regulated in both ICV and PBMC (113). In another study, we recently characterized RNA-seq salivary gland (mandibular and parotid) transcriptome profile in MAP exposed cattle and identified downregulation of genes such as lactoferrin and lactoperoxidase compared to unexposed cattle (114). Using ileal loop model, Khare et al. (115) assessed early transcriptome changes due to MAP infection in the ileal mucosa by RNA-seq and identified downregulated pathways that favor MAP survival (increased mucosal permeability, decreased phagosome-lysosome fusion via inhibition of calcium signaling, and decreased MHC-II expression) and promote persistent infection (increased Th2 response).

RNA-seq has also been employed to study the biology of MAP-MDM interaction and indicated that transcriptome changes in MAP-infected macrophages involve a balance between pro- and anti-inflammatory responses (116), downregulation of phagocytosis and antigen presentation genes, and activation of pathways like suppressor of cytokine signaling (SOCS) and cytokine-inducible SH2-containing protein (CISH) that favor MAP survival (117). More recently, a study by Ariel et al. showed that MAP infection altered gene pathways involved in MDM metabolism, polarization and apoptosis also favoring MAP survival (118). Lastly, using an *in vitro* co-culture model comprised of MAC-T cells and macrophages, Lamont et al. (119) identified activated MAP gene pathways within MAC-T cells that mediate MAP cell wall rebuild and MAP DNA repair to favor the establishment of MAP infection (119).

RNA-seq also has applications in identification of microRNAs (miRNA) for potential use as diagnostic biomarkers of JD. Based on the small RNA-seq method, Farrell et al. (120) identified both known and novel circulating miRNA from the sera of MAP challenged sero-positive and unchallenged control calves at 6 months post-infection. Although between group analysis did not reveal significant differences in miRNA expression, significant differences within group in the levels of miR-205 and miR-432 expression were found between pre-challenge and 6-month post-challenge levels. Using a combination of four circulating serum miRNAs (miR-1976, miR-873-3p, miR-520f-3p, miR-126-3p), Gupta et al. (121) developed a model that can differentiate cattle based on severity of MAP infection as either non-infected, moderately- or severely-infected (121). Liang et al. (122) analyzed both microRNAome (microRNA) and mRNA profiles in calf ileal segments after MAP infection and identified a total of nine differentially expressed miRNAs. Further integrated analyses of miRNAome and the transcriptome indicated that the differentially expressed miRNAs contributed to regulating the host immune response (e.g., proliferation of endothelial cells, bacteria recognition, and regulation of the inflammatory response) to MAP infection. By conducting whole blood miRNA-seq, Malvisi et al. (123) identified 9 and 8 immune response associated miRNAs differentially expressed between infected vs. unexposed and between exposed vs. unexposed cows, respectively. The cows classified as infected were ELISA and fecal culture positive, and both exposed and unexposed cows were ELISA negative but were from JD positive and negative herds, respectively. In another study, three differentially expressed miRNAs were identified in bovine feces with JD diagnostic potential (124). Alongside miRNA, long non-coding RNAs (lncRNA) constitute another type of non-coding RNA that function as regulators of gene expression. In the context of MAP-macrophage interaction, macrophage lncRNAs that regulate immune genes involved in NF-κB2 signaling pathway were identified using RNA-seq (125). RNA-Seq transcriptome studies have enhanced our understanding of host-MAP interaction dynamics. While each RNA-seq studies differ in the kind of tissue studied and the stage of disease in the study animals, the differentially enriched biological pathways identified thus far are predominantly associated with regulation of host immune response to MAP infection. It all begins with the ability of MAP to subvert macrophage response to establish persistent infection followed by altering key immune pathways to promote disease progression.

Before the advent of NGS, transcriptome profiling usually involved microarray hybridization assays where transcript profiling was limited to probes present on the panel (126–129). NGS-based transcriptome profiling enables global transcript analysis and the identification of genes and gene regulation pathways with a potential to identify diagnostic markers and vaccine targets; and this is particularly significant for MAP infection with issues associated with accurate early diagnosis coupled with the absence of an efficacious vaccine (130).

## Genetic Background of Johne's Disease

### Heritability and Breed Susceptibility to MAP Infection in Cattle

Breed susceptibility to MAP infection has been widely reported in cattle indicating genetic predisposition to JD. A survey conducted in the UK reported a higher incidence rate of JD in Channel Island cattle breeds such as Jersey and Guernsey (odds ratio 10.9–12.9) compared to the Holstein breed (131). Similarly, Sorge et al. (132) reported a higher odds ratio (1.4–8.3) in Jersey and Guernsey cattle for being milk ELISA positive for MAP antibodies in comparison with Holsteins, Milking Shorthorn and Brown Swiss breeds. *Bos taurus indicus* (Zebu) animals have a greater odds ratio for being seropositive for MAP-specific antibodies in comparison to *Bos taurus taurus* (Taurine) animals (133). Although the genetic basis of breed susceptibility to MAP infection has not been clearly defined, these reports indicate that JD resistance vary across breeds.

An alternative strategy to reduce or eliminate JD is through genetic selection. Susceptibility to MAP infection is a heritable trait with heritability estimates ranging from 0.03 to 0.228 (49), indicating that there is enough genetic variability for JD resistance to consider selective breeding. The differences in the reported heritability estimates are attributed to factors such as different phenotypes (e.g., results from alternative testing protocols or biological samples), statistical procedures used to determine heritability and variation in JD incidence in the population studied (49). Although low-to-moderate, heritability estimates reflect the role of host genetic makeup in influencing MAP infection status in dairy cattle and offers a potential to employ genetic selection to breed for JD resistance. The need to adopt genetic selection in breeding becomes even more apparent when considering the lack of highly effective treatments and vaccines to deal with JD.

### Genomic Studies Related to Johne's Disease

Further evidence for genetic basis for susceptibility to JD can be found through candidate gene and genome-wide association studies (GWAS). While candidate gene studies look for association between polymorphisms in a particular gene and the disease phenotype, GWAS are conducted at the whole-genome level, usually based on single nucleotide polymorphism (SNP) genetic markers distributed across all chromosomes, statistically tested for their association with a JD phenotype. Recent studies have employed several different phenotypes to identify such associations. To selectively breed for disease resistance, a reliable diagnostic phenotype (e.g., measurement of resistance to MAP infection) must be available to livestock breeders. Given the complex nature of JD and its pathogenesis, potential JD traits can include direct indicators such as MAP load in feces and tissues detected by culture and PCR tests, and indirect indicators such as serum and/or milk ELISA test positivity for MAP-specific antibodies that are indicative of MAP exposure and a subsequent host immune response. As discussed earlier, each available test has its own advantages and disadvantages and they differ in their sensitivities depending on the stage of infection.

A number of GWAS associated with JD in dairy cattle have been published thus far, and several quantitative trait loci (QTLs) and candidate genes have been identified accordingly. Using 12 paternal-half sibling families, Gonda et al. (134) performed genome-wide linkage analysis and identified a QTL located on the *Bos taurus* autosome (BTA) 20 that was associated with MAP infection based on serum culture and/or fecal culture positivity. Using the sire-maternal grandsire model, van Hulzen et al. (135) genotyped 192 Dutch sires and carried out a GWAS using deregressed estimated breeding values (dEBV) for milk ELISA based on records from 265,290 individual Holstein-Friesian cows, and identified five SNPs located on BTA4, BTA5, BTA18, and BTA28 associated with susceptibility to MAP infection. Settles et al. (136) reported QTLs on BTA3 strongly associated with MAP culture positivity in tissues (ileum, ileo-caecal valve and two adjacent ileo-caecal lymph nodes), and QTL on BTA9 associated with presence of MAP in both tissues and feces. By further defining tolerance as both quantitative and categorical trait, Zanella et al. carried out a GWAS using the same data from the Settles et al. study, and identified SNPs located on BTA1, BTA2, BTA6, and BTA15 to be significantly associated with tolerance to MAP infection (137). Further they identified a positional candidate gene *GNA12* located in proximity to significant SNP on BTA 15. Using 50K genotypes from 242 cows, Pant et al. (138) performed a GWAS based on principal component regression analysis (PCA) and identified QTLs on BTA1, BTA5, BTA6, BTA7, BTA10, BTA11, and BTA14 significantly associated with ELISA positivity for MAP antibodies. On BTA7, the positional candidate genes identified included *IL-4, IL-13, IL-5, IRF1, SLC39A3, TNFIP8L1*, and *TICAM1* that are associated with resistance to MAP. In order to fine map the QTLs identified in the Pant et al. study and to identify new QTLs, we further imputed 50K genotypes to the 777K high-density (HD) panel using the FImpute software (139); seven novel QTLs located on BTA15, BTA16, BTA20, and BTA21 were identified in addition to previously reported QTLs on BTA1, BTA5, BTA7, BTA10, BTA11, and BTA14. This indicates that the use of higher density genotyping platforms, or even better whole-genome sequence data, are recommended to perform GWAS for highly polygenic traits such as JD resistance. Furthermore, follow-up bioinformatic analyses revealed several candidate genes involved in pro-inflammatory immune function that are relevant to the host defense against MAP infection: *NLRP3, IFi47, TRIM41, TNFRSF18*, and *TNFRSF4* (140). Alpay et al. has also reported SNPs located on BTA1, BTA2, BTA6, BTA7, BTA17, and BTA29 that are significantly associated with susceptibility to MAP infection in US Holstein cows using a combined phenotype consisting of serum ELISA and fecal MAP culture (141). In an earlier GWAS in Jersey cattle, Zare et al. reported SNPs located on BTA3, BTA16, BTA17, and BTA23 associated with MAP serum ELISA and fecal culture positivity (142). In a GWAS involving 966 Italian Holsteins, Minozzi et al. identified QTLs on BTA9, BTA11, and BTA12 that were associated with serum antibody response (143). In a meta-analysis conducted using two earlier GWAS, Minozzi et al. combined populations from the USA and Italy (*n* = 1,190 cows) and found significant associations on BTA1, BTA6, BTA7, BTA12, BTA13, BTA15, BTA16, BTA21, BTA23, and BTA25 for combined MAP tissue culture and ELISA phenotype (144). Using data from two different populations, Kirkpatrick et al. performed both individual and combined data analysis and identified 51 SNPs located on BTA2, BTA2, BTA4, BTA5, BTA6, BTA7, BTA9, BTA10, BTA13, BTA14, BTA15, BTA16, BTA17, BTA18, BTA20, BTA21, BTA22, BTA23, BTA25, BTA26, and BTA29 that were associated with susceptibility to MAP infection (145). In an across-breed (Holstein and Jersey) GWAS conducted by combining *P*-values from previous independent within-breed GWAS analyses, Sallam et al. identified two significant SNPs Hapmap40994-BTA-46361 and ARS-BFGL-NGS-19381 located on BTA19 and BTA23, respectively (146). Within close proximity to ARS-BFGL-NGS-19381, two positional candidate genes with immunological roles were found including *BTN1A1* (Butyrophilin) and *TDP2* (tyrosyl-DNA phosphodiesterase 2. In a GWAS analyses conducted where JD cases were defined as positive for MAP tissue infection, several positional candidate genes (e.g., *BCAR3, FLVCR2, RASA3, MGC134473, MARK1, C16H1orf115, MARC2, C10H14ORF1*, and *CDC42BPA*) distributed across various chromosomes were identified (147); the putative biological relevance of many of these identified genes involved their roles in processes such as signal transduction, MAP entry into host cells and other immunological effects. In a recent study involving a Chinese Holstein population using a high density SNP panel and two different GWAS methods (GRAMMAR-GC and ROADTRIPS), Gao et al. identified 26 SNPs located on 15 chromosomes to be associated with serum ELISA positivity for MAP infection (148). Interestingly, a few of the positional candidate genes identified in this study were also identified in previous GWAS; this included the genes *IL-4, IL-5, IL-13, IRF1* from the Pant el. study (138) and *TDP2* from the Sallam et al. study (146). In a GWAS involving Canadian Holsteins, SNPs on BTA1, BTA7, BTA9, BTA14, BTA15, BTA17, BTA19, and BTA25 showing significant association with milk ELISA positivity were identified along with two candidate genes, *CD86* and *WNT9B* (49). McGovern et al. conducted a GWAS using imputed sequence data and identified and putative QTLs on BTA1, BTA3, BTA5, BTA6, BTA8, BTA9, BTA10, BTA11, BTA13, BTA14, BTA18, BTA21, BTA23, BTA25, BTA26, BTA27, and BTA29 associated with MAP antibody response (149). They also reported 10 candidate genes harboring these QTLs that have been previously associated with human inflammatory bowel disease. A recently reported sequence-based GWAS in French Holstein and Normande cattle identified three QTLs located on BTA12, BTA13, and BTA23 to be associated with resistance to MAP infection; three functional candidate genes *ABCC4* (BTA12)*, CBFA2T2* (BTA13), and *IER3* (BTA23) that explained a large proportion (28%) of the total additive genetic variance were further identified (150). While causal variants were found within the genes *ABCC4* and *IER3*, the gene *CBFA2T2* is in strong linkage disequilibrium with the causal variant with significant effect. Table 1 lists all the positional candidate genes identified by GWAS.

**Table 1:** Chromosomes with SNPs and candidate genes statistically associated with resistance to Johne's disease in cattle.

**Chr^a^**	**References^b^**	**Candidate genes**
BTA1	(136–138, 141, 144, 149, 151)	*ENSBTAG00000005101, ZBTB20, KARN, LPP, UMPS, SOD1, SSRG, SCL33A1, LDLRAD3,* *KCNAB1, GMPS, TUBA3D*
BTA2	(141, 145, 146)	*CREB1*
BTA3	(136, 142, 145, 147, 149)	*DNAJC6, FOXJ3, EDN2, CTPS,CITED4, NFYC, BCAR3*
BTA4	(135, 145)	*DLD, Q28899, LAMB4, Q6Q146, PNPLA8, LOC784535, Q2YDK7, Q17QP5, EPDR1*
BTA5	(137, 138, 140, 142, 145)	*MANSC4, TRRAP, ALDH1L2, TAS2R42, MAGOHB, KLRA1, KLRJ1, CCDC59,* *TMTC2, FAM113B, AMIGO2, SLC38A4*,
		*SLC38A2, SLC38A1, SFRS2IP, ARID2*,
		*ss61555725, PRICKLE1, PPHLN1, ZCRB1, YAF2, GXYLT1*
		*rs29023629, TMTC1, OVCH1, ERGIC2, FAR2, TM7SF3*
		*CCDC91, PTHLH, KLHDC5, MRPS35, FGFR10P2*
		*PPFIBP1, ARNTL2, STK38L, MED21, ITPR2*
BTA6	(137, 138, 140–142, 144, 145)	*PAPSS1, DKK2, SGMS2, CYP2U1, HADH, LEF1, CRMP1, EVC, EVC2, STX18,* *STK32B, MSX1, CYTL1*
BTA7	(136, 138, 140–142, 144, 145, 148)	*NLRP3, IFI47, OR2B11, TRIM52, GNB2L1, TRIM41, OR2C3, TRIM7, OR2G2,OR2V2, BTNL9,* *COX7B, OR2G3, SPOCK1, IL12B, TIMD4, ITK, SLCO6A1,* *IRF1, IL5, IL13, IL4, SSBP2, ATG10, XRCC4*
BTA8	(136, 143, 147)	*STC1*
BTA9	(136, 143, 145, 149)	*ZDHHC14, PREP, PRDM1*
BTA10	(138, 140, 142, 147, 149)	*SNX1, SOCS4, GCNT3, FAM81A, CCNB2, RORA, ADAM10, ALDH1A2,* *FLVCR2, C10H14ORF1, NARG2, ANXA2, FOXB1, ADAM10, GRINL1A,* *BNIP2, GTF2A2, LIPC, TCF12, MYO1E, CCNB2, RNF111, AQP9,* *GNB5, MYO5C, AP4E1, TRPM7, USP50, USP8, GABPB2, HDC, SLC27A2*
BTA11	(138, 142, 143, 149)	*NPAS2, RNF149, RFX8, TACR1, E2F6, PQLC3, C2o50, KCNF1, PDIA6, ATP6V1C2, NOL10, ODC1, HPCAL1, PRKCE, EPAS1, ATP6V1E2, PIGF, CRIPT, SOCS5, MCFD2, TTC7A, EPCAM, MSH2, KCNK12, MSH6, FBXO11*
BTA12	(143, 144, 147, 150)	*ABCC4, RASA3, MGC134473, GPC6, TYRP2, TGDS, GPR180, SOX21, ABCC4, CFTR/MRP, ABCC4*
BTA13	(142, 144, 145, 149, 150)	*CBFA2T2, SLA2, XKR7*
BTA14	(135, 138, 140, 145, 147, 149)	*CPA6, HAS2, SAMD12, MIR2489, EXT1, TNFRSF11B, EIF3H, UTP23,* *RAD21,AARD,TRPS1, ANGPT1, RABL4, RSPO2, EIF3E, TTC35, TMEM74, TRHR*
BTA15	(135, 137, 140, 141, 144, 145)	*HTR3B, USP28, HTR3A, CLDN25, ZW10, ZBTB16, TMPRSS5, RBM7, REXO2, TTC12, NCAM1,* *KCNA4, FSHB, LOC787432, A5PJ77, CK046, CD44, PAMR1, CACNA1B, LDLRAD3, COMMD9*
BTA16	(135, 137, 140, 142, 144, 145, 147)	*TNFRSF18, TNFRSF4, DNAJC16, CASP9, CELA2A, AGMAT, CTRC, SLC9C2,* *ANKRD45, KLHL20, CENPL, TNNT2, LAD1, TNNI1, CSRP1, TIMM17A,* *SHISA4, MIR2320, TPRG1L, MIR551A, ARHGEF16, PRDM16, PARP1,* *POLR1D, PSEN2, CABC1, SCCPDH, MARK1, C16H1orf115, MARC2, CDC42BPA*
BTA17	(141, 142, 145)	
BTA18	(135, 145, 149)	*ENSBTAG00000040392, TEX101,A2VDX5, IRX5*
BTA19	(135, 141, 146)	*BTN1A1*
BTA20	(134, 135, 140, 145)	*HCN1, EMB, MRPS30, PARP8, SNX18, HSPB3, ESM1, GZMK, ARL15, GZMA,* *CDC20B,GPX8,MIR449A*
BTA21	(135, 136, 140, 144, 145, 147)	*PLD4, KIAA0284, ZBTB42, SIVA1, ADSSL1, INF2, TMEM179, NRAC, BCL2A1,* *ZFAND6, MESDC2, IL16, MCEE*
BTA22	(144, 147, 148)	*ITPR1, CTN4, MyD88*
BTA23	(136, 142, 144–146, 148–150)	*IER3, EEF1E1, SLC17, F13A1, CDYL, PACSIN1, DEF6, TDP2*
BTA24	(145)	
BTA25	(144, 145)	
BTA26	(135, 145, 149)	*PRKG1*
BTA27	(81, 135, 143)	
BTA28	(135)	*ACM3, Q3SX15, ENSBTA00000018960, ZNF25, ZNF334, LOC534200,* *ENSBTAG00000013592, BMS1, RET, CSGALNACT, QCE9S7*

*Adapted and updated from Brito et al. (49)*.

a *Not necessarily the peaks in the same chromosome represents the same genomic region*.

b *Different phenotypes were used in these studies. fecal and/or tissue MAP culture (136, 137); milk ELISA (135); serum ELISA and fecal culture (141); fecal MAP culture and serum ELISA (145); serum ELISA, fecal culture or both (141); MAP in tissues (culture and qPCR) (147); fecal culture or serum ELISA (Population 1) and serum ELISA (Population 2) fecal culture or serum ELISA (146); serum ELISA and/or MAP tissue culture (144); serum ELISA (143, 148); serum ELISA and PCR (150); and, milk/serum ELISA (138, 149): milk/serum ELISA*.

While the above studies made use of different phenotypes, genotyping platforms, populations (e.g., breeds), sample sizes, and employed different analytical models, the identification of numerous QTLs located on almost all chromosomes, but with little overlap, highlights the polygenic and complex nature of JD resistance in dairy cattle (48). It is clear that JD is a complex polygenic trait controlled by a large number of QTLs with small effects distributed across the cattle genome. Although GWAS have been successful in identifying genetic variants that are associated with susceptibility/resistance to MAP infection, the comparison of findings lacks congruency. The possible reasons for this include uncertainty of accurately diagnosing JD owing to issues associated with sensitivity of JD diagnostic methods. Additionally, since JD is a complex chronic disease with multiple stages seen during its pathogenesis, the genetic associations observed during one stage of the disease may differ from others and drawing a conclusive inference is difficult. Additionally, as mentioned above in particular reference to case-control study designs, the methods employed to define the disease phenotype vary, and this could influence the associations that are identified (165).

Candidate gene studies involve studying the association between polymorphisms in a specific gene and a certain phenotype. The candidate genes are selected based on information available in the scientific literature, and their functional role in the pathogenesis of JD, or a similar disease such as CD. Candidate gene studies involve a case-control experimental design where particular candidate gene polymorphisms are genotyped followed by statistical analysis to determine its association with the disease phenotype. Polymorphisms in the candidate genes are typically either SNPs or micro-satellite markers. Genes coding proteins of the immune system are some of the candidate genes that have been studied in the context of JD in cattle. For example, it has been reported that polymorphisms in the following genes: Toll-like receptors (*TLR 1, 2, 4*) (157, 158, 163); Nucleotide binding oligomerization domain containing 2 (*NOD2*) (152–154); Solute carrier family 11 member 1 (*SLC11A1*) (166); Interleukin 10 receptor alpha (*IL-10R*α) (164); SP110 nuclear body protein (*SP110)* (156); and *IFN-*γ*R2, IL-12R*β*1, IL-12R*β*2, IL-23R* (160); Dectin-1 (CLEC7A) (161); Peptidoglycan recognition protein 1 (PGLYRP1) (159); and Wingless-type MMTV integration site family member 2 (*WTN2*) (162) have been significantly associated with MAP infection status in different cattle populations.

In the context of JD, these candidate genes play an active role in immune response acting as either pattern recognition receptors (PRR), receptors of cytokines that drive inflammatory and anti-inflammatory response, or genes that promote killing of intracellular pathogens such as MAP.

*NOD2* (previously known as *CARD 15*) codes for a PRR implicated in recognition of the mycobacterial cell wall constituent, muramyl dipeptide (167). NOD2 further stimulates the transcription factor NF-KB that regulates pro-inflammatory cytokine expression (168). The *TLR1, TLR2*, and *TLR4* genes also code for PRRs that recognize MAP-associated membrane patterns and initiate the host innate and adaptive immune responses in the infected host (169–171). Another PRR coded by *CLEC7A* is expressed on antigen presenting cells (APCs) and is known to recognize MAP and initiate cytokine secretion by phagocytic cells through its synergistic action with TLR2 and TLR4 receptors (169). The *PGLYRP1, SP110*, and *SLC11A1* gene products are also known to trigger innate immune response against intracellular bacteria. While PGLYRP1 functions via neutrophil-mediated killing of bacteria (172), SP110 expression in macrophages is shown to limit mycobacterial replication (173). *SLC11A1*, formerly called as *NRAMP1*, gene product is expressed on phagosomes and is a divalent phagosomal metal ion (Mn^+2^, Fe^+2^) transporter (174) known to control intracellular bacterial replication by regulating divalent ion concentrations within phagosomes (175). The other candidate genes studied for their association with MAP infection status include *IL-10R*α, *IFN-*γ*R2, IL-12R*β*1*/ β*2*, and *IL-23R*, whose gene products serve as the receptors of cytokines IL10, IFN-γ, IL-12, and IL-23, respectively. IFN-γ, IL-12, and IL-23 are pro-inflammatory Th1 cytokines that control MAP infection in the early stages and play a major role in the early cell-mediated immune response driving the cell-mediate immune response in the infected host (89). In contrast, IL-10 is an anti-inflammatory and immunoregulatory cytokine that is involved in regulating the host inflammatory response to MAP infection (176). Another reported candidate gene associated with MAP infection status is the *WNT2* gene with a immunomodulatory functional role in regulating intestinal inflammation and in maintaining tissue homeostasis (177). Not being limited to single SNP associations with MAP infection status, some studies have also reported haplotypes associated with MAP infection status. The list of candidate genes and their polymorphisms/haplotypes significantly associated with MAP infection status are detailed in Table 2.

**Table 2:** List of Johne's disease candidate gene studies in cattle.

**Gene**	**SNP**	**Population**	**Phenotype**	**Location**	**Risk allele**	**Odds ratio**	**References**
NOD2	2197T > C	Holstein, Jersey and Brahman × Angus (*n =* 402)	Serum ELISA, milk PCR, blood PCR, fecal PCR, and fecal culture	Leucine rich domain (non-syn)	C	2.32 (1.41–3.83)	(152)
NOD2	c.^*^1908C>T	Spanish holsteins (*n =* 241)	Fecal culture, fecal PCR, serum ELISA	3′ UTR	C	2.043 (1.22–3.42)	(153)
NOD2	g.521G>A	German holsteins (*n =* 324)	Fecal culture	Exon 4	G	–	(154)
SLC11A1	c.1067C > G	Spanish and Dutch holsteins (*n =* 558)	Fecal culture, PCR, or serum ELISA	Exon 11 (non-syn)	C	1.484 (1.05–2.01)	(155)
	c.1157–91A > T	–	–	Intron 11-12	A	1.592 (0.01–2.3)	
SLC11A1 Haplotype analysis	c.1067C > G and c.1157–91A > T	–	–		CA (risk haplotype)	1.584(1.09–2.3)	
SP110	c.587A>G	Spanish holsteins (*n =* 355)	Fecal culture or serum ELISA	Exon 5 (non-syn)	A	2.7 (1.69-4.54)	(156)
TLR1	G658A (Val220Met)	Slovak spotted cattle Slovak spotted cattle x Holstein Polish red Holstein Pinzgauer Slovakian Simmental Dark brown Carpathians (*n =* 711)	Blood PCR	Ectodomain (non-syn)		3.459	(157)
TLR2	A2038G (Ile680Val)	–	–	Toll/IL-1R domain (non-syn)	–	NA	(157)
TLR4	892G>Y Gly298[Arg,Trp]	–	–	Ectodomain (non-syn)	–	NA	(157)
	G895A (Asp299Asn) G1165A (Gly389Ser) T1167C (Gly389Ser)	– – –	– – –	Ectodomain (non-syn) Ectodomain (non-syn) Ectodomain (non-syn)	– – –	NA NA NA	(157) (157) (157)
TLR2 PGLYRP1 IFNGR2 IL12RB1 IL12RB2 IL23R CLEC7A WNT2	1903 T/C c.480G>A c.1674C>T c.81T>C c.-511A>G c.1417A>C c.589A>G rs43390642: G>T	Dutch holsteins (*n =* 553) Canadian holsteins (*n =* 439) Canadian holsteins (*n =* 439) Canadian holsteins (*n =* 439) Canadian holsteins (*n =* 439) Canadian holsteins (*n =* 439) Canadian holsteins (*n =* 439) German holsteins (*n =* 324)	Fecal culture and ELISA Milk and blood ELISA Milk and blood ELISA Milk and blood ELISA Milk and blood ELISA Milk and blood ELISA Milk and blood ELISA ELISA and fecal culture	LRR (syn) Exon 3 (syn) Coding (syn) Coding (syn) Promoter Coding (non-syn) Exon 5 (non-syn) Promoter	C G T C G C G G	1.7 (1.2–2.8) 1.51 (0.99–2.31) 1.51 (1.03–2.22) 1.62 (1.22–2.15) 1.86 (1.17–2.96) 1.57 (1.01–2.43) 1.42 (1.09–1.9) 2 (1.03–4)	(158) (159) (160) (160) (160) (160) (161) (162)
TLR4 (Haplotype association)	c.-226G>C and c.2021C>T	Canadian holsteins (*n =* 439)	Milk and blood ELISA	c.-226G>C (5' UTR) c.2021C>T (TIR domain/non-syn)	CT (risk haplotype)	1.38	(163)
IL10RA (Haplotype association)	633C > A 984G > A 1185C > T	Canadian holsteins (*n =* 446)	Milk and blood ELISA	All SNPs in coding region (syn)	AGC (risk haplotype)	1.42 (1.06–1.90)	(164)

### Validation of SNPs and Functional Characterization of Candidate Genes

GWAS and candidate gene studies have revealed information on the biology of resistance and genetic basis for JD in cattle. While the genetic influence of MAP infection status is clear and JD is a polygenic disease, validation of all the identified genetic markers based on independent populations as functional genomic studies to uncover the causal mutations are both necessary as well. We recently conducted one such validation study wherein we tested some of the previously identified JD SNPs for their association with sire EBVs estimated for milk ELISA test score (178). Using both General Quasi Likelihood Scoring (GQLS) and single-SNP regression analysis we validated five SNPs (rs41810662, rs41617133, rs110225854, rs110494981, and rs136182707) that offer potential for their inclusion in future marker-assisted breeding programs (178). While our study was limited to a few SNPs, similar validation studies with inclusion of all the reported JD SNPs in the literature should be explored in the future.

JD candidate gene association studies offer great insights on the biological mechanisms involved in JD resistance/susceptibility. However, these are only statistical associations and therefore studies validating the biological significance of JD candidate genes are also warranted. The recent evolution of genetic engineering technologies such as CRISPR/cas9 gene editing (179) can be used to validate JD candidate genes. Using the CRISPR/cas9 gene editing technique, we recently created a *IL10RA* knock-out MAC-T cell line to study functional relevance of candidate gene *IL10RA* (164) in the context of MAP lysate stimulation (101). *IL10RA* functions as a trans-membrane receptor of anti-inflammatory cytokine IL-10 known for its immunoregulatory role during JD immune-pathogenesis. Knocking out *IL10RA* led to dramatic upregulation of pro-inflammatory cytokine expression after stimulation with MAP lysate and further confirmed its role in eliciting anti-inflammatory response via IL-10 during MAP immune response.

## Future Prospects

### Research Tools to Enhance Resolution of Infection Processes

#### Intestinal Organoids

To limit the use of experimental animals and obtain more predictive results, other *in vitro* models that approach biological reality more closely are increasingly being developed. One such model includes organoids that mimic the three-dimensional tissue structure (180). Organoids have been developed using human cells and also in animal models of human disease (181). Relevantly, organoids have also been developed from bovine intestinal tissue (182). Such models can be used to identify host and microbial factors and further characterize early host immune response. Recently, human colon organoids developed from IBD patients were characterized and also tested as a therapeutic model to investigate intestinal healing (183). Going forward, a similar approach to develop intestinal organoids from JD positive cows can be considered to study host-pathogen interaction and immunopathogenesis associated with MAP infection at mucosal surface.

#### Single-Cell RNA-Seq

The macrophage response to MAP infection can be more thoroughly studied *in vitro* using RNA-seq transcriptome profiling (116); however, this also can come with challenges. Post challenge for example, not all macrophages get infected with MAP (63); these ‘by standers’ and cells in heterogeneous physiological states (i.e., cells killing or tolerant to MAP, and apoptotic cells) will lead to a diluted transcription profile that can mask the detection of genes within MAP-infected cells. This limitation, however, can be addressed using single-cell RNA-seq (scRNA-seq) studies. Macrophages can be challenged with florescent MAP followed by sorting infected and uninfected macrophages by fluorescence-activated cell sorting (FACS). The sorted single cells can then be further subjected to RNA-seq transcriptome analysis to identify DEG and enriched biological pathways. Using a similar approach, Saliba et al. studied the macrophage response to *Salmonella* infection and showed how polarization state differed between uninfected (M1) and infected macrophages (M2) (184). In the context of MAP infection, such approach would enable researchers to discern potential differences in the response to MAP challenge between infected and uninfected cells. Understanding host-pathogen interaction using scRNA-seq holds the potential to explore the dynamic changes in host transcriptome profile due to infection at the single-cell level and to further identify biomarkers and to develop novel vaccines and therapeutic targets (185). scRNA-seq is a powerful tool with a wide range applications in basic and medical research fields including the study and control of infectious diseases (186–188).

#### Gene Knock-in Studies

To date, functional validation of biological relevance of JD candidate genes using CRISPR/cas9 has been limited to gene knock-out studies using the non-homologous end-joining (NHEJ) mechanism (101). However, through this approach, the impact of a risk allele in the context of MAP infection cannot be studied. By using a homology-directed (HDR) repair mechanism that relies on addition of a donor template, CRISPR/cas9 gene editing can be further applied to create a mutational homozygous/heterozygous knock-in at a specific SNP loci (189). The selective introduction of mono/bi allelic variants will enable the creation of allelic variant models to compare allele-specific responses for MAP infection (190).

### Integrating GWAS and Transcriptomics—Systems Genetics Approach

Several SNPs or gene mutations have been mapped in the bovine genome for their association with JD through GWAS. However, mapping these genetic variants to underlying molecular biological pathways associated with disease pathogenesis has been largely unsuccessful (191). Understanding this relationship is critical since it could allow for identification and design of therapeutic strategies (192). Integrating GWAS and RNA-seq transcriptomics data using a systems genetics approach has been extensively undertaken to identify loci/variants associated with dysregulated biological network pathways that play a role in complex traits. This integrative systemics genetics approach has been used to identify causal genes and pathways associated with obesity in pigs (193), and mastitis and milk production in cattle (194), and buffaloes (195). As several GWAS and RNA-seq transcriptomic studies are reported for JD, adapting an integrative systems genetics approach will significantly benefit a complex polygenic trait such as JD to identify causal genes and biological pathways that influence disease progression in cattle.

### Vitamin D and MAP Infection

The nutritional status of cattle immediately prior and during the course of infection is likely to be an important determinant not only of disease susceptibility but also on the ability of cattle to control MAP infection. One micronutrient that has attracted considerable attention in that regard is vitamin D. Principally obtained from sunlight, but also from the diet, the availability of vitamin D has increased relevance in terms of housed cattle and deficiency may exacerbate mycobacterial disease susceptibility (196). Additionally, serum vit D deficiency status has been shown to predict tuberculosis (TB) risk in humans in a dose-dependent manner (197). In bovine PBMC studies, 1,25 dihydroxyvitamin D_3_ (1,25-(OH)_2_D_3_) inhibited *M. bovis*-specific IFN-γ production, yet enhanced *M. bovis*-specific nitric oxide (NO) production. Lymphocyte apoptosis was also diminished by addition of 1,25-(OH)_2_D_3_ to PBMC cultures (198). 1,25-(OH)_2_D_3_ was also shown to inhibit the T-cell stimulatory capacity of bovine monocyte-derived dendritic cells (MoDCs) (199). Studies in JD infected cattle are more limited, but vitamin D concentration in cows with positive JD serum ELISA status have been reported to be lower than in cows with a negative status (200). A recent study performed transcriptomic analysis in naturally infected cattle and showed significant differential expression for genes in the vitamin D pathway such as *CYP27A1, CYP27B1, DBP*, and IFNG in JD+ cattle (201). Interestingly, upregulation of *CYP27A1* was observed for cows in subclinical status, whereas the *CYP27B1* expression was enhanced for clinical status cows. Therefore, decreased circulating 1,25-(OH)_2_D_3_ in animals with clinical JD may suggest that these cows have reduced innate immune responses, thereby influencing the ability of animals to fight MAP infection.

### Gut Microbiota and MAP Infection

The composition of gut microbiota and its effect in predisposing humans to IBD is well-documented (202). Chronic inflammation seen in IBD at the gut mucosal level is attributed to reduction in the levels of bacteria with anti-inflammatory properties, as opposed to its pro-inflammatory counterparts (202, 203). This imbalance in gut microbiome diversity commonly referred to as “dysbiosis” has unearthed the significance of gut microbiota in promoting intestinal homeostasis (204). Furthermore, remission of symptoms in CD patients post fecal microbiota transplantation holds promise as a potential alternative therapeutic strategy to immunosuppressive drugs (205). Similar to CD in humans, marked dysbiosis in the fecal microbiota community was observed in MAP-infected cattle when compared to MAP-exposed and MAP-negative cattle (206). Using a rabbit MAP infection model, Arrazuria et al. (207) also noticed changes in gut microbiota composition due to MAP infection and dietary changes. While changes in gut microbiota content during MAP infection are evident, studies linking the same to explain pathogenesis of MAP infection needs to be undertaken. This further holds potential in classifying animals as resistant or susceptible to MAP infection based on their gut microbiota profile. Recent evidence in cattle suggests that rumen microbiome content is heritable (208, 209). Similar understanding of the influence of host genetics on intestinal microbiota could pave the way to selectively manipulate gut microbiota and in turn to breed for JD resistance. Going forward, studies in this regard are highly warranted.

### Genomic Selection for JD Resistance

Genomic selection (GS) involves prediction of breeding values based on whole-genome SNP marker information estimated based on a training population comprising of genotyped animals with measurements for a particular trait(s) (210). Genomic selection is now a routine breeding practice in the dairy cattle industry (as well as many other plant and livestock species). In dairy cattle, the implementation of GS results in substantial reduction of the generation interval, increased genetic progress due to higher selection intensity and greater accuracy of breeding values at an early age, and reduced costs associated with phenotypic data collection on all selection candidates (211). GS has been successfully implemented for various traits in dairy cattle (212), and high genetic gain has been also achieved for lowly-heritable traits (212). Indeed, this is promising for JD. To our best knowledge, no studies have investigated the accuracies of genomic predictions for JD resistance, but this is an area of great importance for future studies.

The success of genomic selection is largely dependent on the size and the design of the training population, which ultimately influence the accuracies of the genomic estimated breeding values (GEBV). However, unlike production traits, implementation of GS for health traits such as JD resistance is complex and poses many challenges (213). Before considering routine genomic evaluations for JD resistance, concerted efforts should be focused on creating a large training population (> 5,000 animals) with accurate JD phenotypic information. As many indicator traits have been used to assess MAP susceptibility in cattle and the associated uncertainty with each trait, a consensus on a trait to be employed in JD selection programs should be arrived first. While measuring MAP load in tissues as an indicator of tolerance projects to be a reliable trait, the need to slaughter animals limits its employment (48, 137). If feasible, it would be worthwhile estimating and comparing GEBV accuracies for each phenotype using different training populations to assess their accuracy for genomic selection for resistance to JD. Consequently, the effect of genetic selection for JD resistance on other traits should also be considered and evaluated.

## Conclusion

The focus of this review was to provide comprehensive update and to highlight recent advancements about JD in cattle from the standpoint of host immune response and genetic regulation of the same. While our focus was from the host perspective, studies understanding the physiology of MAP and their pathogenicity are also happening. Uncovering the role of key immune regulators and genes in JD pathogenesis while also unearthing the impact of host genetic make-up in influencing response to MAP infection has shed immense light on the immunogenetic aspect of JD. Additionally, prior knowledge of host immunogenetic aspects related to an infection is critical in developing vaccines with high immunogenicity as response to vaccines is also determined by the host genetic make-up. With recent advancements in research technologies, our understanding of JD has progressed significantly and will continue to evolve. As we move forward, concerted collaborative efforts will be required to limit the impact of JD on the global livestock industry and on human health.

## Author Contributions

SM, KM, and NK conceptualized the content and overall scope of the review. SM wrote the first draft of the manuscript. LB, SP, and FS provided insights and advice on the genetics and microbiota research input to the review article. All authors contributed to manuscript revision and approved the submitted revision.

## Conflict of Interest

The authors declare that the research was conducted in the absence of any commercial or financial relationships that could be construed as a potential conflict of interest.

## Publisher's Note

All claims expressed in this article are solely those of the authors and do not necessarily represent those of their affiliated organizations, or those of the publisher, the editors and the reviewers. Any product that may be evaluated in this article, or claim that may be made by its manufacturer, is not guaranteed or endorsed by the publisher.

## References

[1] FAO. Meat market review - 2019 outlook. FAO Meat Market Rev. (2019) 1–13.

[2] Wyrzykowski L Reincke K Hemme T. IFCN long-term dairy outlook – the IFCN vision of the dairy world in 2030. In: 19th IFCN Dairy Conference (Cork Ireland).

[3] Barkema HW von Keyserlingk MAG Kastelic JP Lam TJGM Luby C Roy JP. Invited review: changes in the dairy industry affecting dairy cattle health and welfare. J Dairy Sci. (2015) 98:7426–45. 10.3168/jds.2015-9377 26342982

[4] Groeneveld A Peerlings J Bakker M Heijman W. The effect of milk quota abolishment on farm intensity: Shifts and stability. NJAS - Wageningen J Life Sci. (2016) 77:25–37. 10.1016/j.njas.2016.03.003

[5] Thornton PK. Livestock production: recent trends, future prospects. Philos Trans R Soc B Biol Sci. (2010) 365:2853–67. 10.1098/rstb.2010.0134 20713389 PMC2935116

[6] Wapenaar W Archer S Remnant J Murphy A. Control of infectious diseases in dairy cattle. In: Webster J. editor. Achieving Sustainable Production of Milk. Cambridge: Burleigh Dodd Science Publishing. (2017). p. 457–486. 10.19103/AS.2016.0006.23

[7] Rabinowitz PM Kock R Kachani M Kunkel R Thomas J Gilbert J. Toward proof of concept of a one health approach to disease prediction and control. Emerg Infect Dis. (2013) 19:e130265. 10.3201/eid1912.130265 24295136 PMC3840882

[8] Manning EJ Collins MT. *Mycobacterium avium* subsp. paratuberculosis: pathogen, pathogenesis and diagnosis. Revue scientifique et technique. (2001) 20:133–50. 10.20506/rst.20.1.1275 11288509

[9] Whitlock RH Buergelt C. Preclinical and clinical manifestations of paratuberculosis (including pathology). Veterinary Clin North Am Food Animal Practice. (1996) 12:345–56. 10.1016/S0749-0720(15)30410-2 8828109

[10] Whittington R Donat K Weber MF Kelton D Nielsen SS Eisenberg S. Control of paratuberculosis: who, why and how. A review of 48 countries. BMC Vet Res. (2019) 15:198. 10.1186/s12917-019-1943-4 31196162 PMC6567393

[11] Buergelt CD Layton AW Ginn PE Taylor M King JM Habecker PL. The pathology of spontaneous paratuberculosis in the North American bison (Bison bison). Vet Pathol. (2000) 37:428–38. 10.1354/vp.37-5-428 11055866

[12] Chiodini RJ Van Kruiningen HJ. Eastern white-tailed deer as a reservoir of ruminant paratuberculosis. J Am Vet Med Assoc. (1983) 182:168–9. 6826436

[13] Reyes-García R Pérez-de-la-Lastra JM Vicente J Ruiz-Fons F Garrido JM Gortázar C. Large-scale ELISA testing of Spanish red deer for paratuberculosis. Vet Immunol Immunopathol. (2008) 124:75–81. 10.1016/j.vetimm.2008.01.032 18313144

[14] Jessup DA Abbas B Behymer D. Paratuberculosis in tule elk in California. J Am Vet Med Assoc. (1981) 179:1252–4. 7328012

[15] Salgado M Herthnek D Bölske G Leiva S Kruze J. First isolation of *Mycobacterium avium* subsp. paratuberculosis from wild guanacos (Lama guanicoe) on tierra del fuego Island. J Wildl Dis. (2009) 45:295–301. 10.7589/0090-3558-45.2.295 19395739

[16] McClure HM Chiodini RJ Anderson DC Swenson RB Thayer WR Coutu JA. *Mycobacterium paratuberculosis* infection in a colony of stumptail macaques (Macaca Arctoides). J Infect Dis. (1987) 155:1011–9. 10.1093/infdis/155.5.1011 3559275

[17] White CI Birtles RJ Wigley P Jones PH. *Mycobacterium avium* subspecies paratuberculosis in free-living amoebae isolated from fields not used for grazing. Veterinary Rec. (2010) 166:401–2. 10.1136/vr.b4797 20348470

[18] Windsor PA Whittington RJ. Evidence for age susceptibility of cattle to Johne's disease. Veterinary J. (2010) 184:37–44. 10.1016/j.tvjl.2009.01.007 19246220

[19] Sweeney RW. Transmission of paratuberculosis. Veterinary Clin N Am. (1996) 12:305–12. 10.1016/S0749-0720(15)30408-4 8828107

[20] Streeter RN Hoffsis GF Bech-Nielsen S Shulaw WP Rings DM. Isolation of *Mycobacterium paratuberculosis* from colostrum and milk of subclinically infected cows. Am J Vet Res. (1995) 56:1322–4. 8928949

[21] Whittington RJ Windsor PA. In utero infection of cattle with *Mycobacterium avium* subsp. paratuberculosis: a critical review and meta-analysis. Veterinary J. (2009) 179:60–9. 10.1016/j.tvjl.2007.08.023 17928247

[22] Larsen AB Stalheim OH Hughes DE Appell LH Richards WD Himes EM. *Mycobacterium paratuberculosis* in the semen and genital organs of a semen-donor bull. J Am Vet Med Assoc. (1981) 179:169–71. 7263470

[23] Eisenberg SWF Nielen M Santema W Houwers DJ Heederik D Koets AP. Detection of spatial and temporal spread of *Mycobacterium avium* subsp. paratuberculosis in the environment of a cattle farm through bio-aerosols. Vet Microbiol. (2010) 143:284–92. 10.1016/j.vetmic.2009.11.033 20036081

[24] Ott SL Wells SJ Wagner BA. Herd-level economic losses associated with Johne's disease on US dairy operations. Prev Vet Med. (1999) 40:179–92. 10.1016/S0167-5877(99)00037-9 10423773

[25] Tiwari A VanLeeuwen JA Dohoo IR Keefe GP Weersink A. Estimate of the direct production losses in Canadian dairy herds with subclinical *Mycobacterium avium* subspecies paratuberculosis infection. Canad Veterinary J. (2008) 49:569–76. 18624066 PMC2387260

[26] Barrett DJ Good M Hayes M More SJ. The economic impact of Johne's disease in an Irish dairy herd: a case study. Ir Vet J. (2006) 59:282–8.

[27] Shephard RW Williams SH Beckett SD. Farm economic impacts of bovine Johne's disease in endemically infected Australian dairy herds. Aust Vet J. (2016) 94:232–9. 10.1111/avj.12455 27349883

[28] Garcia AB Shalloo L. Invited review: the economic impact and control of paratuberculosis in cattle. J Dairy Sci. (2015) 98:5019–39. 10.3168/jds.2014-9241 26074241

[29] Byrne AW Graham J Milne G Guelbenzu-Gonzalo M Strain S. Is there a relationship between bovine tuberculosis (bTB) herd breakdown risk and *Mycobacterium avium* subsp. paratuberculosis status? An investigation in bTB chronically and non-chronically infected herds. Front Veterinary Sci. (2019) 6:30 10.3389/fvets.2019.00030 30838221 PMC6382694

[30] McNees AL Markesich D Zayyani NR Graham DY. *Mycobacterium paratuberculosis* as a cause of crohn's disease. Expert Rev Gastroenterol Hepatol. (2015) 9:1523–34. 10.1586/17474124.2015.1093931 26474349 PMC4894645

[31] Schwartz D Shafran I Romero C Piromalli C Biggerstaff J Naser N. Use of short-term culture for identication of *Mycobacterium avium* subsp. paratuberculosis in tissue from Cronhs's disease patients. Clin Microbiol Infect. (2000) 6:303–7. 10.1046/j.1469-0691.2000.00093.x 11168138

[32] Sechi LA Scanu AM Molicotti P Cannas S Mura M Dettori G. Detection and isolation of *Mycobacterium avium* subspecies paratuberculosis from intestinal mucosal biopsies of patients with and without Crohn's disease in Sardinia. Am J Gastroenterol. (2005) 100:1529–36. 10.1111/j.1572-0241.2005.41415.x 15984976

[33] Naser SA Schwartz D Shafran I. Isolation of *Mycobacterium avium* subsp paratuberculosis from breast milk of Crohn's disease patients. Am J Gastroenterol. (2000) 95:1094–5. 10.1111/j.1572-0241.2000.01954.x 10763975

[34] Naser SA Ghobrial G Romero C Valentine JF. Culture of *Mycobacterium avium* subspecies paratuberculosis from the blood of patients with Crohn's disease. Lancet. (2004) 364:1039–44. 10.1016/S0140-6736(04)17058-X 15380962

[35] Kirkwood CD Wagner J Boniface K Vaughan J Michalski WP Catto-Smith AG. *Mycobacterium avium* subspecies paratuberculosis in children with early-onset Crohn's disease. Inflamm Bowel Dis. (2009) 15:1643–55. 10.1002/ibd.20967 19462429

[36] Agrawal G Borody T Clancy A Sharma R Huynh R Ramrakha S. Targeted combination antibiotic therapy induces remission in treatment-naïve crohn's disease: a case series. Microorganisms. (2020) 8:371. 10.3390/microorganisms8030371 32155771 PMC7142403

[37] Nacy C Buckley M. Mycobacterium avium Paratuberculosis: Infrequent Human Pathogen or Public Health Threat? Washington, DC: American Academy of Microbiology (2008). 33119237

[38] Rosenfeld G Bressler B. *Mycobacterium avium* paratuberculosis and the etiology of Crohn's disease: a review of the controversy from the clinician's perspective. Canad J Gastroenterol. (2010) 24:619–24. 10.1155/2010/698362 21037992 PMC2975476

[39] Grant IR Foddai ACG Tarrant JC Kunkel B Hartmann FA McGuirk S. Viable *Mycobacterium avium* ssp. paratuberculosis isolated from calf milk replacer. J Dairy Sci. (2017) 100:9723–35. 10.3168/jds.2017-13154 28987590

[40] Collins MT Wells SJ Petrini KR Collins JE Schultz RD Whitlock RH. Evaluation of five antibody detection tests for diagnosis of bovine paratuberculosis. Clin Diagn Lab Immunol. (2005) 12:685–92. 10.1128/CDLI.12.6.685-692.2005 15939741 PMC1151972

[41] Collins MT Gardner IA Garry FB Roussel AJ Wells SJ. Consensus recommendations on diagnostic testing for the detection of paratuberculosis in cattle in the United States. J Am Vet Med Assoc. (2006) 229:1912–19. 10.2460/javma.229.12.1912 17173528

[42] Kalis CHJ Hesselink JW Barkema HW Collins MT. Use of long-term vaccination with a killed vaccine to prevent fecal shedding of *Mycobacterium avium* subsp paratuberculosis in dairy herds. Am J Vet Res. (2001) 62:270–4. 10.2460/ajvr.2001.62.270 11212038

[43] Sweeney RW Whitlock RH Bowersock TL Cleary DL Meinert TR Habecker PL. Effect of subcutaneous administration of a killed *Mycobacterium avium* subsp paratuberculosis vaccine on colonization of tissues following oral exposure to the organism in calves. Am J Vet Res. (2009) 70:493–7. 10.2460/ajvr.70.4.493 19335105

[44] Bannantine JP Hines ME Bermudez LE Talaat AM Sreevatsan S Stabel JR. A rational framework for evaluating the next generation of vaccines against *Mycobacterium avium* subspecies paratuberculosis. Front Cell Infect Microbiol. (2014) 4:126. 10.3389/fcimb.2014.00126 25250245 PMC4158869

[45] Lu Z Mitchell RM Smith RL Van Kessel JS Chapagain PP Schukken YH. The importance of culling in Johne's disease control. J Theor Biol. (2008) 254:135–46. 10.1016/j.jtbi.2008.05.008 18573505

[46] Fecteau ME. Paratuberculosis in Cattle. Veterinary Clin N Am - Food Animal Pract. (2018) 34:209–22. 10.1016/j.cvfa.2017.10.011 29275033

[47] Geraghty T Graham DA Mullowney P More SJ. A review of bovine Johne's disease control activities in 6 endemically infected countries. Prev Vet Med. (2014) 116:1–1. 10.1016/j.prevetmed.2014.06.003 24997766

[48] Kirkpatrick BW Shook GE. Genetic susceptibility to paratuberculosis. Veterinary Clin N A Food Animal Pract. (2011) 27:559–71. 10.1016/j.cvfa.2011.07.003 22023834

[49] Brito LF Mallikarjunappa S Sargolzaei M Koeck A Chesnais J Schenkel FS. The genetic architecture of milk ELISA scores as an indicator of Johne's disease (paratuberculosis) in dairy cattle. J Dairy Sci. (2018) 101:10062–75. 10.3168/jds.2017-14250 30219422

[50] Stinson KJ Baquero MM Plattner BL. Resilience to infection by *Mycobacterium avium* subspecies paratuberculosis following direct intestinal inoculation in calves. Vet Res. (2018) 49:1–2. 10.1186/s13567-018-0553-7 30001739 PMC6044094

[51] Momotani E Whipple DL Thiermann AB Cheville NF. Role of M cells and macrophages in the entrance of *Mycobacterium paratuberculosis* into Domes of Ileal Peyer's patches in calves. Vet Pathol. (1988) 25:131–7. 10.1177/030098588802500205 3363791

[52] Sigurardóttir ÓG Press CM Evensen O. Uptake of *Mycobacterium avium* subsp. paratuberculosis through the distal small intestinal mucosa in goats: an ultrastructural study. Veterinary Pathol. (2001) 38:184–9. 10.1354/vp.38-2-184 11280374

[53] Secott TE Lin TL Wu CC. Fibronectin attachment protein is necessary for efficient attachment and invasion of epithelial cells by *Mycobacterium avium* subsp. paratuberculosis. Infect Immun. (2002) 70:2670–5. 10.1128/IAI.70.5.2670-2675.2002 11953410 PMC127902

[54] Ponnusamy D Periasamy S Tripathi BN Pal A. *Mycobacterium avium* subsp. paratuberculosis invades through M cells and enterocytes across ileal and jejunal mucosa of lambs. Res Vet Sci. (2013) 94:306–12. 10.1016/j.rvsc.2012.09.023 23122809

[55] Coussens P Lamont EA Kabara E Sreevatsan S. “Host-pathogen interactions and intracellular survival of *Mycobacterium avium* subsp. paratuberculosis. In: Paratuberculosis: Organism, Disease, Control. Wallingford: CABI (2020). p. 109–25. 10.1079/9781845936136.0109

[56] Schlesinger LS Bellinger-Kawahara CG Payne NR Horwitz MA. Phagocytosis of *Mycobacterium tuberculosis* is mediated by human monocyte complement receptors and complement component C3. J Immunol. (1990) 144:2771–80. 2108212

[57] Astarie-Dequeker C N'Diaye EN Le Cabec V Rittig MG Prandi J Maridonneau-Parini I. The mannose receptor mediates uptake of pathogenic and nonpathogenic mycobacteria and bypasses bactericidal responses in human macrophages. Infect Immun. (1999) 67:469–77. 10.1128/IAI.67.2.469-477.1999 9916047 PMC96343

[58] Schlesinger LS. Macrophage phagocytosis of virulent but not attenuated strains of *Mycobacterium tuberculosis* is mediated by mannose receptors in addition to complement receptors. J Immunol. (1993) 150:2920–30. 8454864

[59] Souza CD Evanson OA Sreevatsan S Weiss DJ. Cell membrane receptors on bovine mononuclear phagocytes involved in phagocytosis of *Mycobacterium avium* subsp paratuberculosis. Am J Vet Res. (2007) 68:975–80. 10.2460/ajvr.68.9.975 17764412

[60] Peterson PK Gekker G Hu S Sheng WS Anderson WR Ulevitch RJ. CD14 receptor-mediated uptake of nonopsonized *Mycobacterium tuberculosis* by human microglia. Infect Immun. (1995) 63:1598–602. 10.1128/iai.63.4.1598-1602.1995 7534279 PMC173196

[61] Arsenault RJ Maattanen P Daigle J Potter A Griebel P Napper S. From mouth to macrophage: mechanisms of innate immune subversion by *Mycobacterium avium* subsp. Paratuberculosis. Vet Res. (2014) 45:54. 10.1186/1297-9716-45-54 24885748 PMC4046017

[62] Hostetter J Steadham E Haynes J Bailey T Cheville N. Phagosomal maturation and intracellular survival of *Mycobacterium avium* subspecies paratuberculosis in J774 cells. Comp Immunol Microbiol Infect Dis. (2003) 26:269–83. 10.1016/S0147-9571(02)00070-X 12676127

[63] Kabara E Coussens PM. Infection of primary bovine macrophages with *Mycobacterium avium* subspecies paratuberculosis suppresses host cell apoptosis. Front Microbiol. (2012) 3:215. 10.3389/fmicb.2012.00215 22833736 PMC3400940

[64] Weiss DJ Evanson OA McClenahan DJ Abrahamsen MS Walcheck BK. Regulation of expression of major histocompatibility antigens by bovine macrophages infected with *Mycobacterium avium* subsp. paratuberculosis or *Mycobacterium avium subsp*. avium. Infect Immun. (2001) 69:1002–8. 10.1128/IAI.69.2.1002-1008.2001 11159996 PMC97980

[65] Sommer S Pudrith CB Colvin CJ Coussens PM. *Mycobacterium avium* subspecies paratuberculosis suppresses expression of IL-12p40 and iNOS genes induced by signalling through CD40 in bovine monocyte-derived macrophages. Vet Immunol Immunopathol. (2009) 128:44–52. 10.1016/j.vetimm.2008.10.294 19022505

[66] Arsenault RJ Li Y Bell K Doig K Potter A Griebel PJ. *Mycobacterium avium* subsp. paratuberculosis inhibits gamma interferon-induced signaling in bovine monocytes: Insights into the cellular mechanisms of Johne's disease. Infect Immun. (2012) 80:3039–48. 10.1128/IAI.00406-12 22689821 PMC3418731

[67] Coussens PM. *Mycobacterium paratuberculosis* and the bovine immune system. Anim Health Res Rev. (2001) 2:141–61. 10.1079/AHRR200134 11831436

[68] Sweeney RW. Pathogenesis of Paratuberculosis. Veterinary Clin N Am Food Animal Pract. (2011) 27:537–46. 10.1016/j.cvfa.2011.07.001 22023832

[69] Stabel JR. Host responses to *Mycobacterium avium* subsp. paratuberculosis: a complex arsenal. Anim Health Res Rev. (2006) 7:61–70. 10.1017/S1466252307001168 17389054

[70] Jungersen G Huda A Hansen JJ Lind P. Interpretation of the gamma interferon test for diagnosis of subclinical paratuberculosis in cattle. Clin Diagn Lab Immunol. (2002) 9:453–60. 10.1128/CDLI.9.2.453-460.2002 11874893 PMC119921

[71] Zhao B Collins MT Czuprynski CJ. Effects of gamma interferon and nitric oxide on the interaction of *Mycobacterium avium* subsp. paratuberculosis with bovine monocytes. Infect Immun. (1997) 65:1761–66. 10.1128/iai.65.5.1761-1766.1997 9125559 PMC175213

[72] Hostetter J Huffman E Byl K Steadham E. Inducible nitric oxide synthase immunoreactivity in the granulomatous intestinal lesions of naturally occurring bovine Johne's disease. Vet Pathol. (2005) 42:241–9. 10.1354/vp.42-3-241 15872370

[73] Khalifeh MS Al-Majali AM Stabel JR. Role of nitric oxide production in dairy cows naturally infected with *Mycobacterium avium* subsp. paratuberculosis. Vet Immunol Immunopathol. (2009) 131:97–104. 10.1016/j.vetimm.2009.03.020 19409621

[74] Clarke CJ. The pathology and pathogenesis of paratuberculosis in ruminants and other species. J Comp Pathol. (1997) 116:217–61. 10.1016/S0021-9975(97)80001-1 9147244

[75] DeKuiper JL Cooperider HE Lubben N Ancel CM Coussens PM. *Mycobacterium avium* subspecies paratuberculosis drives an innate Th17-Like T cell response regardless of the presence of antigen-presenting cells. Front Veterinary Sci. (2020) 7:108. 10.3389/fvets.2020.00108 32258066 PMC7089878

[76] Khader SA Bell GK Pearl JE Fountain JJ Rangel-Moreno J Cilley GE. IL-23 and IL-17 in the establishment of protective pulmonary CD4+ T cell responses after vaccination and during *Mycobacterium tuberculosis* challenge. Nat Immunol. (2007) 8:369–77. 10.1038/ni1449 17351619

[77] Roussey JA Oliveira LJ Langohr IM Sledge DG Coussens PM. Regulatory T cells and immune profiling in johne's disease lesions. Vet Immunol Immunopathol. (2016) 181:39–50. 10.1016/j.vetimm.2016.03.008 27013348

[78] Hines ME Stabel JR Sweeney RW Griffin F Talaat AM Bakker D. Experimental challenge models for Johne's disease: a review and proposed international guidelines. Vet Microbiol. (2007) 122:197–222. 10.1016/j.vetmic.2007.03.009 17467201

[79] Mikkelsen H Jungersen G Nielsen SS. Association between milk antibody and interferon-gamma responses in cattle from *Mycobacterium avium* subsp. paratuberculosis infected herds. Vet Immunol Immunopathol. (2009) 127:235–41. 10.1016/j.vetimm.2008.10.315 19027177

[80] Pollock JM Welsh MD. The WC1+ γδ T-cell population in cattle: a possible role in resistance to intracellular infection. Vet Immunol Immunopathol. (2002) 89:105–14. 10.1016/S0165-2427(02)00200-3 12383642

[81] Guerra-Maupome M Slate JR McGill JL. Gamma delta T cell function in ruminants. Veterinary Clin N Am Food Animal Pract. (2019) 35:453–69. 10.1016/j.cvfa.2019.08.001 31590897

[82] Plattner BL Hostetter JM. Comparative Gamma Delta T cell immunology: a focus on mycobacterial disease in cattle. Vet Med Int. (2011) 2011:1–8. 10.4061/2011/214384 21647391 PMC3103839

[83] Plattner BL Doyle RT Hostetter JM. Gamma-delta T cell subsets are differentially associated with granuloma development and organization in a bovine model of mycobacterial disease. Int J Exp Pathol. (2009) 90:587–97. 10.1111/j.1365-2613.2009.00679.x 19758417 PMC2803249

[84] Baquero MM Plattner BL. Bovine WC1+ γδ T lymphocytes modify monocyte-derived macrophage responses during early *Mycobacterium avium* subspecies paratuberculosis infection. Vet Immunol Immunopathol. (2015) 170:65–72. 10.1016/j.vetimm.2015.12.002 26848050

[85] Baquero MM. Bovine WC1+ and WC1neg γδ T Lymphocytes influence monocyte differentiation and monocyte-derived dendritic cell maturation during *in vitro Mycobacterium avium* subspecies paratuberculosis infection. Front Immunol. (2017) 8:534. 10.3389/fimmu.2017.00534 28588573 PMC5439176

[86] Baquero MM Plattner BL. Bovine peripheral blood WC1+ and WC1neg γδ T lymphocytes modulate monocyte-derived macrophage effector functions during *in vitro Mycobacterium avium* subspecies paratuberculosis infection. Cell Immunol. (2017) 315:34–44. 10.1016/j.cellimm.2017.01.009 28284486

[87] Sweeney RW Jones DE Habecker P Scott P. Interferon-γ and interleukin 4 gene expression in cows infected with *Mycobacterium paratuberculosis*. Am J Vet Res. (1998) 59:842–7. 9659548

[88] Khalifeh MS Stabel JR. Upregulation of transforming growth factor-beta and interleukin-10 in cows with clinical Johne's disease. Vet Immunol Immunopathol. (2004) 99:39–46. 10.1016/j.vetimm.2004.01.009 15113652

[89] Coussens PM Verman N Coussens MA Elftman MD McNulty AM. Cytokine gene expression in peripheral blood mononuclear cells and tissues of cattle infected with *Mycobacterium avium* subsp. paratuberculosis: evidence for an inherent proinflammatory gene expression pattern. Infect Immun. (2004) 72:1409–22. 10.1128/IAI.72.3.1409-1422.2004 14977946 PMC356024

[90] Stabel JR. Cytokine secretion by peripheral blood mononuclear cells from cows infected with *Mycobacterium paratuberculosis*. Am J Vet Res. (2000) 61:754–60. 10.2460/ajvr.2000.61.754 10895895

[91] Koets AP Rutten VPMG De Boer M Bakker D Valentin-Weigand P Van Eden W. Differential changes in heat shock protein-, lipoarabinomannan-, and purified protein derivative-specific immunoglobulin G1 and G2 isotype responses during bovine *Mycobacterium avium* subsp. paratuberculosis infection. Infect Immun. (2001) 69:1492–8. 10.1128/IAI.69.3.1492-1498.2001 11179318 PMC98047

[92] de Almeida DE Colvin CJ Coussens PM. Antigen-specific regulatory T cells in bovine paratuberculosis. Vet Immunol Immunopathol. (2008) 125:234–45. 10.1016/j.vetimm.2008.05.019 18602164

[93] Coussens PM Sipkovsky S Murphy B Roussey J Colvin CJ. Regulatory T cells in cattle and their potential role in bovine paratuberculosis. Comp Immunol Microbiol Infect Dis. (2012) 35:233–9. 10.1016/j.cimid.2012.01.004 22285689

[94] Roussey JA Steibel JP Coussens PM. Regulatory T Cell activity and signs of T cell unresponsiveness in bovine paratuberculosis. Front Veterinary Sci. (2014) 1:20. 10.3389/fvets.2014.00020 26664919 PMC4668878

[95] Koets A Rutten V Hoek A Van Mil F Müller K Bakker D. Progressive bovine paratuberculosis is associated with local loss of CD4+ T cells, increased frequency of γδ T cells, and related changes in T-cell function. Infect Immun. (2002) 70:3856–64. 10.1128/IAI.70.7.3856-3864.2002 12065529 PMC128076

[96] Plattner BL Chiang YW Roth JA Platt R Huffman E Zylstra J. Direct inoculation of *Mycobacterium avium* subspecies paratuberculosis into ileocecal peyer's patches results in colonization of the intestine in a calf model. Vet Pathol. (2011) 48:584–92. 10.1177/0300985810383874 20930105

[97] Weiss DJ Evanson OA de Souza C Abrahamsen MS. A critical role of interleukin-10 in the response of bovine macrophages to infection by *Mycobacterium avium* subsp paratuberculosis. Am J Vet Res. (2005) 66:721–6. 10.2460/ajvr.2005.66.721 15900955

[98] Buza JJ Hikono H Mori Y Nagata R Hirayama S Bari AM. Neutralization of interleukin-10 Significantly enhances gamma interferon expression in peripheral blood by stimulation with Johnin purified protein derivative and by infection with *Mycobacterium avium* subsp. paratuberculosis in experimentally infected cat. Infect Immun. (2004) 72:2425–8. 10.1128/IAI.72.4.2425-2428.2004 15039374 PMC375198

[99] Souza CD Evanson OA Weiss DJ. Role of the mitogen-activated protein kinase pathway in the differential response of bovine monocytes to *Mycobacterium avium* subsp. paratuberculosis and *Mycobacterium avium subsp. avium*. Microbes Infect. (2007) 9:1545–52. 10.1016/j.micinf.2007.08.008 18035573

[100] Souza CD Evanson OA Weiss DJ. Role of cell membrane receptors in the suppression of monocyte anti-microbial activity against *Mycobacterium avium* subsp. paratuberculosis. Microb Pathog. (2008) 44:215–23. 10.1016/j.micpath.2007.09.006 18079089

[101] Mallikarjunappa S Shandilya UK Sharma A Lamers K Bissonnette N Karrow NA. Functional analysis of bovine interleukin-10 receptor alpha in response to *Mycobacterium avium* subsp. paratuberculosis lysate using CRISPR/Cas9. BMC Genet. (2020) 21:121. 10.1186/s12863-020-00925-4 33138773 PMC7607837

[102] Fair KL Colquhoun J Hannan NRF. Intestinal organoids for modelling intestinal development and disease. Philos Trans R Soc B Biol Sci. (2018) 373:20170217. 10.1098/rstb.2017.0217 29786552 PMC5974440

[103] Khare S Nunes JS Figueiredo JF Lawhon SD Rossetti CA Gull T. Early phase morphological lesions and transcriptional responses of bovine ileum infected with *Mycobacterium avium* subsp. paratuberculosis. Vet Pathol. (2009) 46:717–28. 10.1354/vp.08-VP-0187-G-FL 19276052

[104] Wu CW Livesey M Schmoller SK Manning EJB Steinberg H Davis WC. Invasion and persistence of *Mycobacterium avium* subsp. paratuberculosis during early stages of Johne's disease in calves. Infect Immun. (2007) 75:2110–9. 10.1128/IAI.01739-06 17296749 PMC1865790

[105] Allen AJ Park KT Barrington GM Lahmers KK Hamilton MJ Davis WC. Development of a bovine ileal cannulation model to study the immune response and mechanisms of pathogenesis of paratuberculosis. Clin Vaccine Immunol. (2009) 16:453–63. 10.1128/CVI.00347-08 19225077 PMC2668272

[106] Koo HC Park YH Hamilton MJ Barrington GM Davies CJ Kim JB. Analysis of the immune response to *Mycobacterium avium* subsp. paratuberculosis in experimentally infected calves. Infect Immun. (2004) 72:6870–83. 10.1128/IAI.72.12.6870-6883.2004 15557608 PMC529129

[107] Purdie AC Plain KM Begg DJ de Silva K Whittington RJ. Expression of genes associated with the antigen presentation and processing pathway are consistently regulated in early *Mycobacterium avium* subsp. paratuberculosis infection. Comp Immunol Microbiol Infect Dis. (2012) 35:151–62. 10.1016/j.cimid.2011.12.007 22239946

[108] Thirunavukkarasu S Plain KM de Silva K Begg D Whittington RJ Purdie AC. Expression of genes associated with cholesterol and lipid metabolism identified as a novel pathway in the early pathogenesis of *Mycobacterium avium* subspecies paratuberculosis-infection in cattle. Vet Immunol Immunopathol. (2014) 160:147–57. 10.1016/j.vetimm.2014.04.002 24930699

[109] Stinson KJ Duffield TF Kelton DF Baquero MM Plattner BL. A preliminary study investigating effects of oral monensin sodium in an enteric *Mycobacterium avium* ssp. paratuberculosis infection model of calves. J Dairy Sci. (2019) 102:9097–106. 10.3168/jds.2018-15980 31400899

[110] Charavaryamath C Gonzalez-Cano P Fries P Gomis S Doig K Scruten E. Host responses to persistent *Mycobacterium avium* subspecies paratuberculosis infection in surgically isolated bovine ileal segments. Clin Vaccine Immunol. (2013) 20:156–65. 10.1128/CVI.00496-12 23221000 PMC3571287

[111] Begg DJ Plain KM de Silva K Gurung R Gunn A Purdie AC. Immunopathological changes and apparent recovery from infection revealed in cattle in an experimental model of Johne's disease using a lyophilised culture of *Mycobacterium avium* subspecies paratuberculosis. Vet Microbiol. (2018) 219:53–62. 10.1016/j.vetmic.2018.03.029 29778205

[112] Hempel RJ Bannantine JP Stabel JR. Transcriptional profiling of ileocecal valve of holstein dairy cows infected with *Mycobacterium avium* subsp. paratuberculosis. PLoS ONE. (2016) 11:e0153932. 10.1371/journal.pone.0153932 27093613 PMC4836751

[113] Alonso-Hearn M Canive M Blanco-Vazquez C Torremocha R Balseiro A Amado J. RNA-Seq analysis of ileocecal valve and peripheral blood from Holstein cattle infected with *Mycobacterium avium* subsp. paratuberculosis revealed dysregulation of the CXCL8/IL8 signaling pathway. Sci Rep. (2019) 9:14845. 10.1038/s41598-019-51328-0 31619718 PMC6795908

[114] Mallikarjunappa S Adnane M Cormican P Karrow NA Meade KG. Characterization of the bovine salivary gland transcriptome associated with *Mycobacterium avium* subsp. paratuberculosis experimental challenge. BMC Genomics. (2019) 20:491. 10.1186/s12864-019-5845-4 31195975 PMC6567491

[115] Khare S Drake KL Lawhon SD Nunes JES Figueiredo JF Rossetti CA. Systems analysis of early host gene expression provides clues for transient *Mycobacterium avium* ssp avium vs. persistent *Mycobacterium avium* ssp paratuberculosis intestinal infections. PLoS ONE. (2016) 11:e0161946. 10.1371/journal.pone.0161946 27653506 PMC5031438

[116] Casey ME Meade KG Nalpas NC Taraktsoglou M Browne JA Killick KE. Analysis of the bovine monocyte-derived macrophage response to *Mycobacterium avium* subspecies paratuberculosis infection using RNA-seq. Front Immunol. (2015) 6:23. 10.3389/fimmu.2015.00023 25699042 PMC4316787

[117] Marino R Capoferri R Panelli S Minozzi G Strozzi F Trevisi E. Johne's disease in cattle: an *in vitro* model to study early response to infection of *Mycobacterium avium* subsp. paratuberculosis using RNA-seq. Mol Immunol. (2017) 91:259–271. 10.1016/j.molimm.2017.08.017 28988040

[118] Ariel O Gendron D Dudemaine PL Gévry N Ibeagha-Awemu EM Bissonnette N. Transcriptome profiling of bovine macrophages infected by *Mycobacterium avium* spp. paratuberculosis depicts foam cell and innate immune tolerance phenotypes. Front Immunol. (2020) 10:2874. 10.3389/fimmu.2019.02874 31969876 PMC6960179

[119] Lamont EA Xu WW Sreevatsan S. Host-*Mycobacterium avium* subsp. paratuberculosis interactome reveals a novel iron assimilation mechanism linked to nitric oxide stress during early infection. BMC Genomics. (2013) 14:694. 10.1186/1471-2164-14-694 24112552 PMC3832399

[120] Farrell D Shaughnessy RG Britton L MacHugh DE Markey B Gordon S V. The identification of circulating MiRNA in bovine serum and their potential as novel biomarkers of early *Mycobacterium avium* subsp paratuberculosis infection. PLoS ONE. (2015) 10:e0134310. 10.1371/journal.pone.0134310 26218736 PMC4517789

[121] Gupta SK Maclean PH Ganesh S Shu D Buddle BM Wedlock DN. Detection of microRNA in cattle serum and their potential use to diagnose severity of Johne's disease. J Dairy Sci. (2018) 101:10259–270. 10.3168/jds.2018-14785 30197143

[122] Liang G Malmuthuge N Guan Y Ren Y Griebel PJ Guan LL. Altered microRNA expression and pre-mRNA splicing events reveal new mechanisms associated with early stage *Mycobacterium avium* subspecies paratuberculosis infection. Sci Rep. (2016) 6:24964. 10.1038/srep24964 27102525 PMC4840452

[123] Malvisi M Palazzo F Morandi N Lazzari B Williams JL Pagnacco G. Responses of bovine innate immunity to *Mycobacterium avium* subsp. Paratuberculosis infection revealed by changes in gene expression levels of MicroRNA. PLoS ONE. (2016) 11:e0164461. 10.1371/journal.pone.0164461 27760169 PMC5070780

[124] Shaughnessy RG Farrell D Stojkovic B Browne JA Kenny K Gordon S V. Identification of microRNAs in bovine faeces and their potential as biomarkers of Johne's disease. Sci Rep. (2020) 10:5908. 10.1038/s41598-020-62843-w 32246047 PMC7125074

[125] Gupta P Peter S Jung M Lewin A Hemmrich-Stanisak G Franke A. Analysis of long non-coding RNA and mRNA expression in bovine macrophages brings up novel aspects of *Mycobacterium avium* subspecies paratuberculosis infections. Sci Rep. (2019) 9:1571. 10.1038/s41598-018-38141-x 30733564 PMC6367368

[126] Coussens PM Pudrith CB Skovgaard K Ren X Suchyta SP Stabel JR. Johne's disease in cattle is associated with enhanced expression of genes encoding IL-5, GATA-3, tissue inhibitors of matrix metalloproteinases 1 and 2, and factors promoting apoptosis in peripheral blood mononuclear cells. Vet Immunol Immunopathol. (2005) 105:221–34. 10.1016/j.vetimm.2005.02.009 15808302

[127] Aho AD McNulty AM Coussens PM. enhanced expression of interleukin-1α and tumor necrosis factor receptor-associated protein 1 in ileal tissues of cattle infected with *Mycobacterium avium* subsp. paratuberculosis. Infect Immun. (2003) 71:6479–86. 10.1128/IAI.71.11.6479-6486.2003 14573670 PMC219597

[128] Skovgaard K Grell SN Heegaard PMH Jungersen G Pudrith CB Coussens PM. Differential expression of genes encoding CD30L and P-selectin in cattle with Johne's disease: Progress toward a diagnostic gene expression signature. Vet Immunol Immunopathol. (2006) 112:210–24. 10.1016/j.vetimm.2006.02.006 16621022

[129] Verschoor CP Pant SD You Q Kelton DF Karrow NA. Gene expression profiling of PBMCs from Holstein and Jersey cows sub-clinically infected with *Mycobacterium avium* ssp. paratuberculosis. Vet Immunol Immunopathol. (2010) 137:1–1. 10.1016/j.vetimm.2010.03.026 20447698

[130] van den Esker MH Koets AP. Application of transcriptomics to enhance early diagnostics of mycobacterial infections, with an emphasis on *Mycobacterium avium* ssp. paratuberculosis. Veterinary Sci. (2019) 6:59. 10.3390/vetsci6030059 31247942 PMC6789504

[131] Çetinkaya B Erdogan H Morgan K. Relationships between the presence of Johne's disease and farm and management factors in dairy cattle in England. Prev Vet Med. (1997) 32:253–66. 10.1016/S0167-5877(97)00028-7 9443332

[132] Sorge US Lissemore K Godkin A Hendrick S Wells S Kelton D. Associations between paratuberculosis milk ELISA result, milk production, and breed in Canadian dairy cows. J Dairy Sci. (2011) 94:754–61. 10.3168/jds.2010-3404 21257043

[133] Roussel AJ Libal MC Whitlock RL Hairgrove TB Barling KS Thompson JA. Prevalence of and risk factors for paratuberculosis in purebred beef cattle. J Am Vet Med Assoc. (2005) 226:773–8. 10.2460/javma.2005.226.773 15776952

[134] Gonda MG Kirkpatrick BW Shook GE Collins MT. Identification of a QTL on BTA20 affecting susceptibility to *Mycobacterium avium* ssp. paratuberculosis infection in US Holsteins. Anim Genet. (2007) 38:389–96. 10.1111/j.1365-2052.2007.01627.x 17617211

[135] van Hulzen KJE Schopen GCB van Arendonk JAM Nielen M Koets AP Schrooten C. Genome-wide association study to identify chromosomal regions associated with antibody response to *Mycobacterium avium* subspecies paratuberculosis in milk of Dutch Holstein-Friesians. J Dairy Sci. (2012) 95:2740–8. 10.3168/jds.2011-5005 22541504

[136] Settles M Zanella R McKay SD Schnabel RD Taylor JF Whitlock R. A whole genome association analysis identifies loci associated with *Mycobacterium avium* subsp. paratuberculosis infection status in US holstein cattle. Anim Genet. (2009) 40:655–62. 10.1111/j.1365-2052.2009.01896.x 19422364

[137] Zanella R Settles ML McKay SD Schnabel R Taylor J Whitlock RH. Identification of loci associated with tolerance to Johne's disease in Holstein cattle. Anim Genet. (2011) 42:28–38. 10.1111/j.1365-2052.2010.02076.x 20477805

[138] Pant SD Schenkel FS Verschoor CP You Q Kelton DF Moore SS. A principal component regression based genome wide analysis approach reveals the presence of a novel QTL on BTA7 for MAP resistance in holstein cattle. Genomics. (2010) 95:176–82. 10.1016/j.ygeno.2010.01.001 20060464

[139] Sargolzaei M Chesnais JP Schenkel FS. A new approach for efficient genotype imputation using information from relatives. BMC Genomics. (2014) 15:478. 10.1186/1471-2164-15-478 24935670 PMC4076979

[140] Mallikarjunappa S Sargolzaei M Brito LF Meade KG Karrow NA Pant SD. Short communication: uncovering quantitative trait loci associated with resistance to *Mycobacterium avium* ssp. paratuberculosis infection in Holstein cattle using a high-density single nucleotide polymorphism panel. J Dairy Sci. (2018) 101:7280–6. 10.3168/jds.2018-14388 29753465

[141] Alpay F Zare Y Kamalludin MH Huang X Shi X Shook GE. Genome-wide association study of susceptibility to infection by *Mycobacterium avium* subspecies paratuberculosis in holstein cattle. PLoS ONE. (2014) 9:e111704. 10.1371/journal.pone.0111704 25473852 PMC4256300

[142] Zare Y Shook GE Collins MT Kirkpatrick BW. Genome-wide association analysis and genomic prediction of *Mycobacterium avium* subspecies paratuberculosis infection in US Jersey cattle. PLoS ONE. (2014) 9:e88380. 10.1371/journal.pone.0088380 24523889 PMC3921184

[143] Minozzi G Buggiotti L Stella A Strozzi F Luini M Williams JL. Genetic loci involved in antibody response to *Mycobacterium* avium ssp. paratuberculosis in cattle. PLoS ONE. (2010) 5:e11117. 10.1371/journal.pone.0011117 20559561 PMC2886106

[144] Minozzi G Williams JL Stella A Strozzi F Luini M Settles ML. Meta-analysis of two genome-wide association studies of bovine paratuberculosis. PLoS ONE. (2012) 7:e32578. 10.1371/journal.pone.0032578 22396781 PMC3292576

[145] Kirkpatrick BW Shi X Shook GE Collins MT. Whole-Genome association analysis of susceptibility to paratuberculosis in Holstein cattle. Anim Genet. (2011) 42:149–60. 10.1111/j.1365-2052.2010.02097.x 20618184

[146] Sallam AM Zare Y Alpay F Shook GE Collins MT Alsheikh S. An across-breed genome wide association analysis of susceptibility to paratuberculosis in dairy cattle. J Dairy Res. (2017) 84:61–7. 10.1017/S0022029916000807 28252359

[147] Kiser JN White SN Johnson KA Hoff JL Taylor JF Neibergs HL. Identification of loci associated with susceptibility to *Mycobacterium avium* subspecies paratuberculosis (Map) tissue infection in cattle. J Anim Sci. (2017) 95:1080–91. 10.2527/jas.2016.1152 28380509

[148] Gao Y Jiang J Yang S Cao J Han B Wang Y. Genome-wide association study of *Mycobacterium avium* subspecies paratuberculosis infection in Chinese holstein. BMC Genomics. (2018) 19:972. 10.1186/s12864-018-5385-3 30591025 PMC6307165

[149] McGovern SP Purfield DC Ring SC Carthy TR Graham DA Berry DP. Candidate genes associated with the heritable humoral response to *Mycobacterium avium* ssp. paratuberculosis in dairy cows have factors in common with gastrointestinal diseases in humans. J Dairy Sci. (2019) 102:4249–4263. 10.3168/jds.2018-15906 30852025

[150] Sanchez M-P Guatteo R Davergne A Saout J Grohs C Deloche M-C. Identification of the ABCC4, IER3, and CBFA2T2 candidate genes for resistance to paratuberculosis from sequence-based GWAS in Holstein and Normande dairy cattle. Genet Select Evolut. (2020) 52:14. 10.1186/s12711-020-00535-9 32183688 PMC7077142

[151] Zare Y Shook GE Collins MT Kirkpatrick BW. Short communication: heritability estimates for susceptibility to *Mycobacterium avium* subspecies paratuberculosis infection defined by ELISA and fecal culture test results in Jersey cattle. J Dairy Sci. (2014) 97:4562–7. 10.3168/jds.2013-7426 24819128

[152] Pinedo PJ Buergelt CD Donovan GA Melendez P Morel L Wu R. Association between CARD15/NOD2 gene polymorphisms and paratuberculosis infection in cattle. Vet Microbiol. (2009) 134:346–52. 10.1016/j.vetmic.2008.09.052 18926647

[153] Ruiz-Larrañaga O Garrido JM Iriondo M Manzano C Molina E Koets AP. Genetic association between bovine NOD2 polymorphisms and infection by *Mycobacterium avium* subsp. paratuberculosis in Holstein-Friesian cattle. Anim Genet. (2010) 41:652–5. 10.1111/j.1365-2052.2010.02055.x 20477790

[154] Küpper JD Brandt HR Erhardt G. Genetic association between NOD2 polymorphism and infection status by *Mycobacterium avium* ssp. paratuberculosis in German Holstein cattle. Anim Genet. (2014) 45:114–6. 10.1111/age.12097 24320212

[155] Ruiz-Larrañaga O Garrido JM Manzano C Iriondo M Molina E Gil A. Identification of single nucleotide polymorphisms in the bovine solute carrier family 11 member 1 (SLC11A1) gene and their association with infection by *Mycobacterium avium* subspecies paratuberculosis. J Dairy Sci. (2010) 93:1713–21. 10.3168/jds.2009-2438 20338449

[156] Ruiz-Larrañaga O Garrido JM Iriondo M Manzano C Molina E Montes I. SP110 as a novel susceptibility gene for *Mycobacterium avium* subspecies paratuberculosis infection in cattle. J Dairy Sci. (2010) 93:5950–8. 10.3168/jds.2010-3340 21094769

[157] Mucha R Bhide MR Chakurkar EB Novak M Mikula I. Toll-like receptors TLR1, TLR2 and TLR4 gene mutations and natural resistance to *Mycobacterium avium* subsp. paratuberculosis infection in cattle. Vet Immunol Immunopathol. (2009) 128:381–8. 10.1016/j.vetimm.2008.12.007 19131114

[158] Koets A Santema W Mertens H Oostenrijk D Keestra M Overdijk M. Susceptibility to paratuberculosis infection in cattle is associated with single nucleotide polymorphisms in Toll-like receptor 2 which modulate immune responses against *Mycobacterium avium* subspecies paratuberculosis. Prev Vet Med. (2010) 93:305–15. 10.1016/j.prevetmed.2009.11.008 20005587

[159] Pant SD Verschoor CP Schenkel FS You Q Kelton DF Karrow NA. Bovine PGLYRP1 polymorphisms and their association with resistance to *Mycobacterium avium* ssp. Paratuberculosis. Anim Genet. (2011) 42:354–60. 10.1111/j.1365-2052.2010.02153.x 21749417

[160] Pant SD Verschoor CP Skelding AM Schenkel FS You Q Biggar GA. Bovine IFNGR2, IL12RB1, IL12RB2, and IL23R polymorphisms and MAP infection status. Mammalian Genome. (2011) 22:583–8. 10.1007/s00335-011-9332-8 21597988

[161] Pant SD Verschoor CP Schenkel FS You Q Kelton DF Karrow NA. Bovine CLEC7A genetic variants and their association with seropositivity in Johne's disease ELISA. Gene. (2014) 537:302–7. 10.1016/j.gene.2013.12.020 24393710

[162] Pauciullo A Küpper J Brandt H Donat K Iannuzzi L Erhardt G. Wingless-type MMTV integration site family member 2 (WNT2) gene is associated with resistance to MAP in faecal culture and antibody response in Holstein cattle. Anim Genet. (2015) 46:122–32. 10.1111/age.12261 25643727

[163] Sharma BS Abo-Ismail MK Schenkel FS You Q Verschoor CP Pant SD. Association of TLR4 polymorphisms with *Mycobacterium avium* subspecies paratuberculosis infection status in Canadian Holsteins. Anim Genet. (2015) 46:560–5. 10.1111/age.12333 26360329

[164] Verschoor CP Pant SD You Q Schenkel FS Kelton DF Karrow NA. Polymorphisms in the gene encoding bovine interleukin-10 receptor alpha are associated with *Mycobacterium avium* ssp. paratuberculosis infection status. BMC Genet. (2010) 11:23. 10.1186/1471-2156-11-23 20398313 PMC2873551

[165] Küpper J Brandt H Donat K Erhardt G. Phenotype definition is a main point in genome-wide association studies for bovine *Mycobacterium avium* ssp. paratuberculosis infection status. Animal. (2014) 8:1586–93. 10.1017/S1751731114001232 25231280

[166] Pinedo PJ Buergelt CD Donovan GA Melendez P Morel L Wu R. Candidate gene polymorphisms (BoIFNG, TLR4, SLC11A1) as risk factors for paratuberculosis infection in cattle. Prev Vet Med. (2009) 91:189–96. 10.1016/j.prevetmed.2009.05.020 19525022

[167] Girardin SE Boneca IG Viala J Chamaillard M Labigne A Thomas G. Nod2 is a general sensor of peptidoglycan through Muramyl Dipeptide (MDP) detection. J Biol Chem. (2003) 278:8869–72. 10.1074/jbc.C200651200 12527755

[168] Abbott DW Wilkins A Asara JM Cantley LC. The Crohn's disease protein, NOD2, requires RIP2 in order to induce ubiquitinylation of a novel site on NEMO. Curr Biol. (2004) 14:2217–27. 10.1016/j.cub.2004.12.032 15620648

[169] Ferwerda G Kullberg BJ de Jong DJ Girardin SE Langenberg DML van Crevel R. *Mycobacterium paratuberculosis* is recognized by Toll-like receptors and NOD2. J Leukoc Biol. (2007) 82:1011–8. 10.1189/jlb.0307147 17652449

[170] Byun E-H Kim WS Kim J-S Won C-J Choi H-G Kim H-J. *Mycobacterium paratuberculosis* CobT activates dendritic cells via engagement of toll-like receptor 4 resulting in Th1 cell expansion. J Biol Chem. (2012) 287:38609–24. 10.1074/jbc.M112.391060 23019321 PMC3493906

[171] Park H-S Back YW Son Y-J Kim H-J. *Mycobacterium avium* subsp. paratuberculosis MAP1889c protein induces maturation of dendritic cells and drives Th2-biased immune responses. Cells. (2020) 9:944. 10.3390/cells9040944 32290379 PMC7226993

[172] Cho JH Fraser IP Fukase K Kusumoto S Fujimoto Y Stahl GL. Human peptidoglycan recognition protein S is an effector of neutrophil-mediated innate immunity. Blood. (2005) 106:2551–8. 10.1182/blood-2005-02-0530 15956276 PMC1895263

[173] Wu Y Guo Z Yao K Miao Y Liang S Liu F. The transcriptional foundations of Sp110-mediated macrophage (RAW264.7) resistance to *Mycobacterium tuberculosis* H37Ra. Sci Rep. (2016) 6:22041. 10.1038/srep22041 26912204 PMC4766572

[174] Soe-Lin S Sheftel AD Wasyluk B Ponka P. Nramp1 equips macrophages for efficient iron recycling. Exp Hematol. (2008) 36:929–937. 10.1016/j.exphem.2008.02.013 18456389

[175] Govoni G Gros P. Macrophage NRAMP1 and its role in resistance to microbial infections. Inflam Res. (1998) 47:277–84. 10.1007/s000110050330 9719491

[176] Hussain T Shah SZA Zhao D Sreevatsan S Zhou X. The role of IL-10 in *Mycobacterium avium* subsp. paratuberculosis infection. Cell Commun Signal. (2016) 14:1–4. 10.1186/s12964-016-0152-z 27905994 PMC5131435

[177] Liu X Lu R Wu S Zhang Y Xia Y Sartor BR. Wnt2 inhibits enteric bacterial-induced inflammation in intestinal epithelial cells. Inflamm Bowel Dis. (2012) 18:418–29. 10.1002/ibd.21788 21674728 PMC3294455

[178] Mallikarjunappa S Schenkel FS Brito LF Bissonnette N Miglior F Chesnais J. Association of genetic polymorphisms related to Johne's disease with estimated breeding values of Holstein sires for milk ELISA test scores. BMC Vet Res. (2020) 16:165. 10.1186/s12917-020-02381-9 32460776 PMC7254716

[179] Singh V Braddick D Dhar PK. Exploring the potential of genome editing CRISPR-Cas9 technology. Gene. (2017) 599:1–18. 10.1016/j.gene.2016.11.008 27836667

[180] Schutgens F Clevers H. Human organoids: tools for understanding biology and treating diseases. Ann Rev Pathol. (2020) 15:211–34. 10.1146/annurev-pathmechdis-012419-032611 31550983

[181] Fonseca KL Rodrigues PNS Olsson IAS Saraiva M. Experimental study of tuberculosis: from animal models to complex cell systems and organoids. PLoS Pathog. (2017) 13:e1006421. 10.1371/journal.ppat.1006421 28817682 PMC5560521

[182] Hamilton CA Young R Jayaraman S Sehgal A Paxton E Thomson S. Development of *in vitro* enteroids derived from bovine small intestinal crypts. Vet Res. (2018) 49:54. 10.1186/s13567-018-0547-5 29970174 PMC6029049

[183] d'Aldebert E Quaranta M Sébert M Bonnet D Kirzin S Portier G. Characterization of human colon organoids from inflammatory bowel disease patients. Front Cell Dev Biol. (2020) 8:363. 10.3389/fcell.2020.00363 32582690 PMC7287042

[184] Saliba A-E Li L Westermann AJ Appenzeller S Stapels DAC Schulte LN. Single-cell RNA-seq ties macrophage polarization to growth rate of intracellular Salmonella. Nat Microbiol. (2017) 2:16206. 10.1038/nmicrobiol.2016.206 27841856

[185] Penaranda C Hung DT. Single-cell RNA sequencing to understand host–pathogen interactions. ACS Infect Dis. (2019) 5:336–44. 10.1021/acsinfecdis.8b00369 30702856

[186] Tang X Huang Y Lei J Luo H Zhu X. The single-cell sequencing: new developments and medical applications. Cell Biosci. (2019) 9:53. 10.1186/s13578-019-0314-y 31391919 PMC6595701

[187] Yasen A Aini A Wang H Li W Zhang C Ran B. Progress and applications of single-cell sequencing techniques. Infect Genet Evolut. (2020) 80:104198. 10.1016/j.meegid.2020.104198 31958516

[188] Lin WN Tay MZ Lu R Liu Y Chen CH Cheow LF. The role of single-cell technology in the study and control of infectious diseases. Cells. (2020) 9:1–28. 10.3390/cells9061440 32531928 PMC7348906

[189] Paquet D Kwart D Chen A Sproul A Jacob S Teo S. Efficient introduction of specific homozygous and heterozygous mutations using CRISPR/Cas9. Nature. (2016) 533:125–9. 10.1038/nature17664 27120160

[190] Wu J Tang B Tang Y. Allele-specific genome targeting in the development of precision medicine. Theranostics. (2020) 10:3118–37. 10.7150/thno.43298 32194858 PMC7053192

[191] Gallagher MD Chen-Plotkin AS. The post-GWAS era: from association to function. Am J Human Genet. (2018) 102:717–30. 10.1016/j.ajhg.2018.04.002 29727686 PMC5986732

[192] Moreno-Moral A Petretto E. From integrative genomics to systems genetics in the rat to link genotypes to phenotypes. Dis Model Mech. (2016) 9:1097–110. 10.1242/dmm.026104 27736746 PMC5087832

[193] Kogelman LJA Zhernakova DV Westra H-J Cirera S Fredholm M Franke L. An integrative systems genetics approach reveals potential causal genes and pathways related to obesity. Genome Med. (2015) 7:105. 10.1186/s13073-015-0229-0 26482556 PMC4617184

[194] Fang L Sahana G Su G Yu Y Zhang S Lund MS. Integrating Sequence-based GWAS and RNA-Seq provides novel insights into the genetic basis of mastitis and milk production in dairy cattle. Sci Rep. (2017) 7:45560. 10.1038/srep45560 28358110 PMC5372096

[195] Deng T Liang A Liang S Ma X Lu X Duan A. Integrative analysis of transcriptome and GWAS data to identify the hub genes associated with milk yield trait in buffalo. Front Genet. (2019) 10:36. 10.3389/fgene.2019.00036 30804981 PMC6371051

[196] Greenstein RJ Su L Brown ST. Vitamins A & D inhibit the growth of mycobacteria in radiometric culture. PLoS ONE. (2012) 7:e29631. 10.1371/journal.pone.0029631 22235314 PMC3250462

[197] Aibana O Huang C-C Aboud S Arnedo-Pena A Becerra MC Bellido-Blasco JB. Vitamin D status and risk of incident tuberculosis disease: a nested case-control study, systematic review, and individual-participant data meta-analysis. PLoS Med. (2019) 16:e1002907. 10.1371/journal.pmed.1002907 31509529 PMC6738590

[198] Waters WR Nonnecke BJ Rahner TE Palmer MV Whipple DL Horst RL. Modulation of Mycobacterium bovis-specific responses of bovine peripheral blood mononuclear cells by 1,25-dihydroxyvitamin D3. Clin Diagn Lab Immunol. (2001) 8:1204–12. 10.1128/CDLI.8.6.1204-1212.2001 11687464 PMC96250

[199] Corripio-Miyar Y Mellanby RJ Morrison K McNeilly TN. 1,25-Dihydroxyvitamin D3 modulates the phenotype and function of Monocyte derived dendritic cells in cattle. BMC Veterinary Res. (2017) 13:390. 10.1186/s12917-017-1309-8 29237505 PMC5729451

[200] Sorge US Molitor T Linn J Gallaher D Wells SW. Cow-level association between serum 25-hydroxyvitamin D concentration and *Mycobacterium avium* subspecies paratuberculosis antibody seropositivity: a pilot study. J Dairy Sci. (2013) 96:1030–7. 10.3168/jds.2012-5929 23261386

[201] Stabel JR Reinhardt TA Hempel RJ. Short communication: Vitamin D status and responses in dairy cows naturally infected with *Mycobacterium avium* ssp. paratuberculosis. J Dairy Sci. (2019) 102:1594–600. 10.3168/jds.2018-15241 30594355

[202] Nishida A Inoue R Inatomi O Bamba S Naito Y Andoh A. Gut microbiota in the pathogenesis of inflammatory bowel disease. Clin J Gastroenterol. (2018) 11:1–10. 10.1007/s12328-017-0813-5 29285689

[203] Frank DN St Amand AL Feldman RA Boedeker EC Harpaz N Pace NR. Molecular-phylogenetic characterization of microbial community imbalances in human inflammatory bowel diseases. Proc Natl Acad Sci USA. (2007) 104:13780–5. 10.1073/pnas.0706625104 17699621 PMC1959459

[204] Belizário JE Faintuch J. Microbiome and gut dysbiosis. Exp Suppl. (2018) 109:459–76. 10.1007/978-3-319-74932-7_13 30535609

[205] Sokol H Landman C Seksik P Berard L Montil M Nion-Larmurier I. Fecal microbiota transplantation to maintain remission in Crohn's disease: a pilot randomized controlled study. Microbiome. (2020) 8:12. 10.1186/s40168-020-0792-5 32014035 PMC6998149

[206] Fecteau M-E Pitta DW Vecchiarelli B Indugu N Kumar S Gallagher SC. Dysbiosis of the fecal microbiota in cattle infected with *Mycobacterium avium* subsp. paratuberculosis. PLoS ONE. (2016) 11:e0160353. 10.1371/journal.pone.0160353 27494144 PMC4975387

[207] Arrazuria R Elguezabal N Juste RA Derakhshani H Khafipour E. *Mycobacterium avium* subspecies paratuberculosis infection modifies gut microbiota under different dietary conditions in a rabbit model. Front Microbiol. (2016) 7:446. 10.3389/fmicb.2016.00446 27065994 PMC4815054

[208] Li F Li C Chen Y Liu J Zhang C Irving B. Host genetics influence the rumen microbiota and heritable rumen microbial features associate with feed efficiency in cattle. Microbiome. (2019) 7:92. 10.1186/s40168-019-0699-1 31196178 PMC6567441

[209] Abbas W Howard JT Paz HA Hales KE Wells JE Kuehn LA. Influence of host genetics in shaping the rumen bacterial community in beef cattle. Sci Rep. (2020) 10:15101. 10.1038/s41598-020-72011-9 32934296 PMC7493918

[210] Meuwissen THE Hayes BJ Goddard ME. Prediction of total genetic value using genome-wide dense marker maps. Genetics. (2001) 157:1819–29. 10.1093/genetics/157.4.1819 11290733 PMC1461589

[211] Schaeffer LR. Strategy for applying genome-wide selection in dairy cattle. J Animal Breed Genet. (2006) 123:218–23. 10.1111/j.1439-0388.2006.00595.x 16882088

[212] García-Ruiz A Cole JB VanRaden PM Wiggans GR Ruiz-López FJ Van Tassell CP. Changes in genetic selection differentials and generation intervals in US Holstein dairy cattle as a result of genomic selection. Proc Natl Acad Sci USA. (2016) 113:E3995–4004. 10.1073/pnas.1519061113 27354521 PMC4948329

[213] Hu G Do DN Gray J Miar Y. Selection for favorable health traits: a potential approach to cope with diseases in farm animals. Animals. (2020) 10:1717. 10.3390/ani10091717 32971980 PMC7552752

